# Supplementary material for: Triiodothyronine (T3) promotes brown fat hyperplasia via thyroid hormone receptor α mediated adipocyte progenitor cell proliferation
Source: Nat Commun. 2022 Jun 13;13:3394. doi: 10.1038/s41467-022-31154-1 (PMC9192766; doi:10.1038/s41467-022-31154-1)
Supplement: Supplementary file 1 — Supplementary Information [file 41467_2022_31154_MOESM1_ESM.pdf]

Supplementary information

Triiodothyronine (T3) promotes brown fat hyperplasia via thyroid hormone receptor α mediated adipocyte progenitor cell proliferation

Liu *et al*

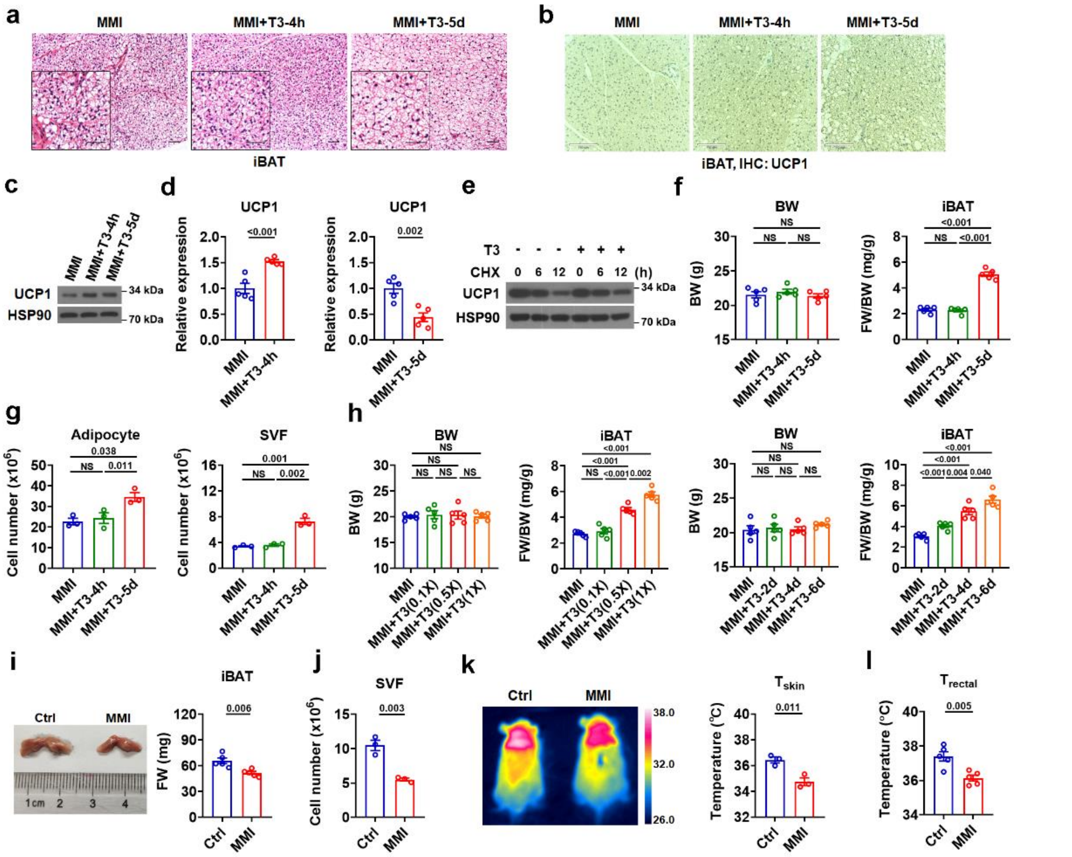

Supplementary Figure 1. T3 increases thermogenic capacity by promoting iBAT expansion.

(a) Representative H&E staining of iBAT from hypothyroid (MMI) mice and MMI mice treated with T3 for 4 h or 5 d. Scale bar, 100 μm. (b) Representative UCP1 staining of iBAT from MMI mice and MMI mice treated with T3 for 4 h or 5 d. Scale bar, 110 μm. (c) Western blot analysis of UCP1 in the iBAT of MMI mice and MMI mice treated with T3 for 4 h or 5 d (related to Fig. 1d). (d) qRT-PCR analysis of *Ucp1* in the iBAT of MMI mice and MMI mice treated with T3 for 4 h or 5 d (n = 5). (e) Western blot of UCP1 in mature iBAT-SVF-derived adipocytes treated with cycloheximide (CHX) and T3 for 0, 6, and 12 h as indicated. (f) Body weight (BW) and ratio of fat weight (FW) to BW (FW/BW) for iBAT of MMI mice and MMI mice treated with T3 for 4 h or 5 d (n = 5). (g) Number of mature adipocytes (left) and SVF cells (right) of iBAT per mouse in MMI mice and MMI mice with different T3 treatments (n = 3). iBAT was minced and then digested for 20 min. (h) BW and FW/BW for iBAT of MMI mice and MMI mice with daily T3 injection for 4 d at indicated doses (n = 5) or for indicated days at 0.5X standard dose (n = 5). (i) Representative photograph and FW of iBAT from euthyroid (Ctrl) and MMI mice (n = 5). (j) Number of stromal vascular fraction (SVF) cells of iBAT per mouse in Ctrl and MMI mice (n = 3). (k) Representative infrared images and quantification of average dorsal skin temperature (T_{skin}) of Ctrl and MMI mice (n = 3). (l) Rectal temperature (T_{rectal}) of Ctrl and MMI mice (n = 5). Means ± SEM are shown. *P* values were calculated by two-tailed unpaired Student's *t* test for d, f-l. NS, not significant. Source data are provided as a Source Data file.

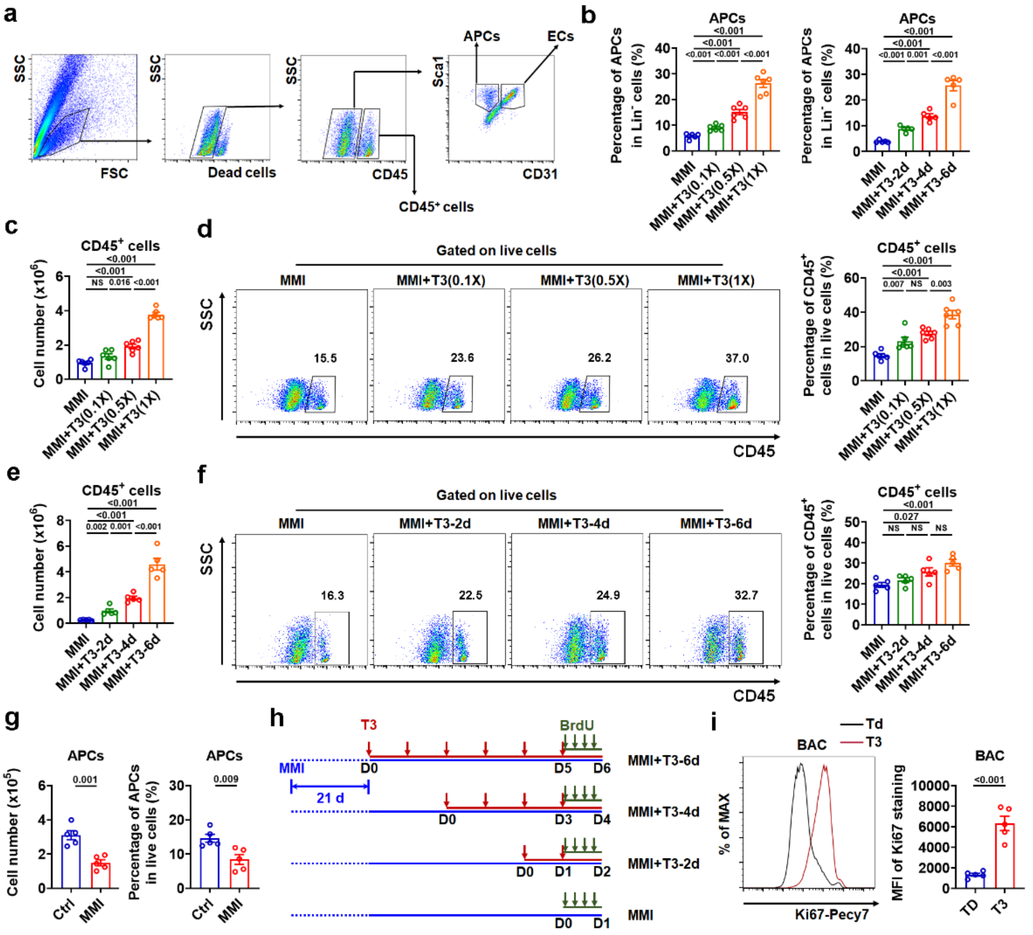

Supplementary Figure 2. T3 expands adipocyte progenitor population in iBAT depot.

(a) Representative flow cytometry dot plots indicating sequential gating used to identify adipocyte progenitor cells (APCs) from iBAT depot. (b) Percentage of APCs in Lin^{-} cells (CD45^{-} cells) of iBAT in MMI mice and MMI mice with daily T3 injection for 4 d at indicated doses (left, n = 6) or for indicated days at 0.5X standard dose (right, n = 5). (c-f) Number of CD45^{+} cells in iBAT per mouse (c and e), FACS plot (d and f, left) and percentage of CD45^{+} cells in live SVF cells of iBAT (d and f, right) in MMI mice and MMI mice with daily T3 injection for 4 d at indicated doses (n = 6) or for indicated days at 0.5X standard dose (n = 5). (g) Number and percentage of APCs in iBAT per mouse in Ctrl and MMI mice (n = 5). (h) Schematic of the BrdU labeling experiment. (i) Representative flow cytometry histograms and quantification of Ki67 expression in brown adipocyte precursor cell line (BAC) in the absence or presence of T3 (n = 5). Means ± SEM are shown. *P* values were calculated by two-tailed unpaired Student's *t* test for b-g, and i. NS, not significant. Source data are provided as a Source Data file.

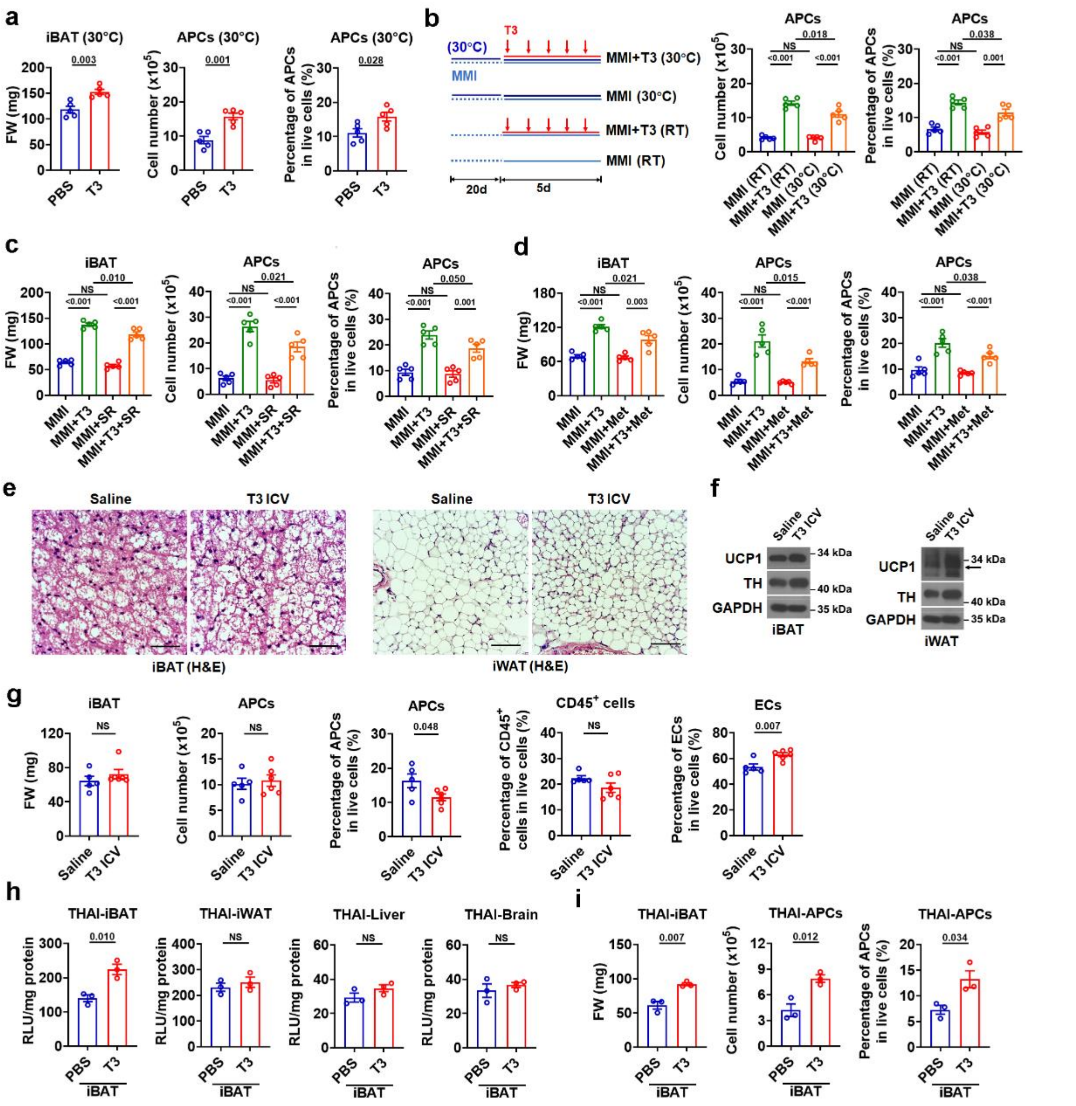

Supplementary Figure 3. T3 promotes APC proliferation in BAT depot in a cell-autonomous manner.

(a) FW of iBAT, number of APCs in iBAT per mouse and percentage of APCs in live SVF cells of iBAT from wild-type C57BL/6 (WT) mice housed at 30°C with daily T3 injection for 5 d (n = 5). (b) Schematic of T3 treatment (MMI+T3) of MMI mice housed at room temperature (RT) or 30°C (left). Number of APCs in iBAT per mouse and percentage of APCs in live SVF cells in iBAT from MMI mice housed at RT or 30°C with daily T3 injection for 5 d (right, n = 5). (c) FW of iBAT, number of APCs in iBAT per mouse and percentage of APCs in live SVF cells of iBAT from MMI mice with daily SR59230A (SR) or T3 injection for 5 d (n = 5). (d) FW of iBAT, number of APCs in iBAT per mouse and percentage of APCs in live SVF cells of iBAT from MMI mice with daily metoprolol (Met) or T3 injection for 5 d (n = 5). (e-f) Representative H&E staining (e) and western blot analysis of UCP1 and tyrosine hydroxylase (TH). Scale bar, 100 μm. (f) of iBAT and iWAT from WT mice following intracerebroventricular (ICV) injection of saline or T3 for 5 d. (g) FW of iBAT, number of APCs in iBAT per mouse, and percentage of APCs, $CD45^+$ cells and endothelial cells in live SVF cells of iBAT from WT mice receiving ICV administration of saline (n = 5) or T3 (n = 6) for 5 d . (h) Luciferase activity in tissue samples (iBAT, iWAT, liver, and brain) from thyroid hormone action indicator (THAI) mice receiving local injection of T3 to iBAT region for 5 d through rolling microneedles (n = 3). (i) FW of iBAT, number of APCs in iBAT per mouse, and percentage of APCs in live SVF cells of iBAT from THAI mice with local injection of T3 to iBAT region (n = 3). Means ± SEM are shown. *P* values

were calculated by two-tailed unpaired Student's *t* test for a-d, g-i. NS, not significant. Source data are provided as a Source Data file.

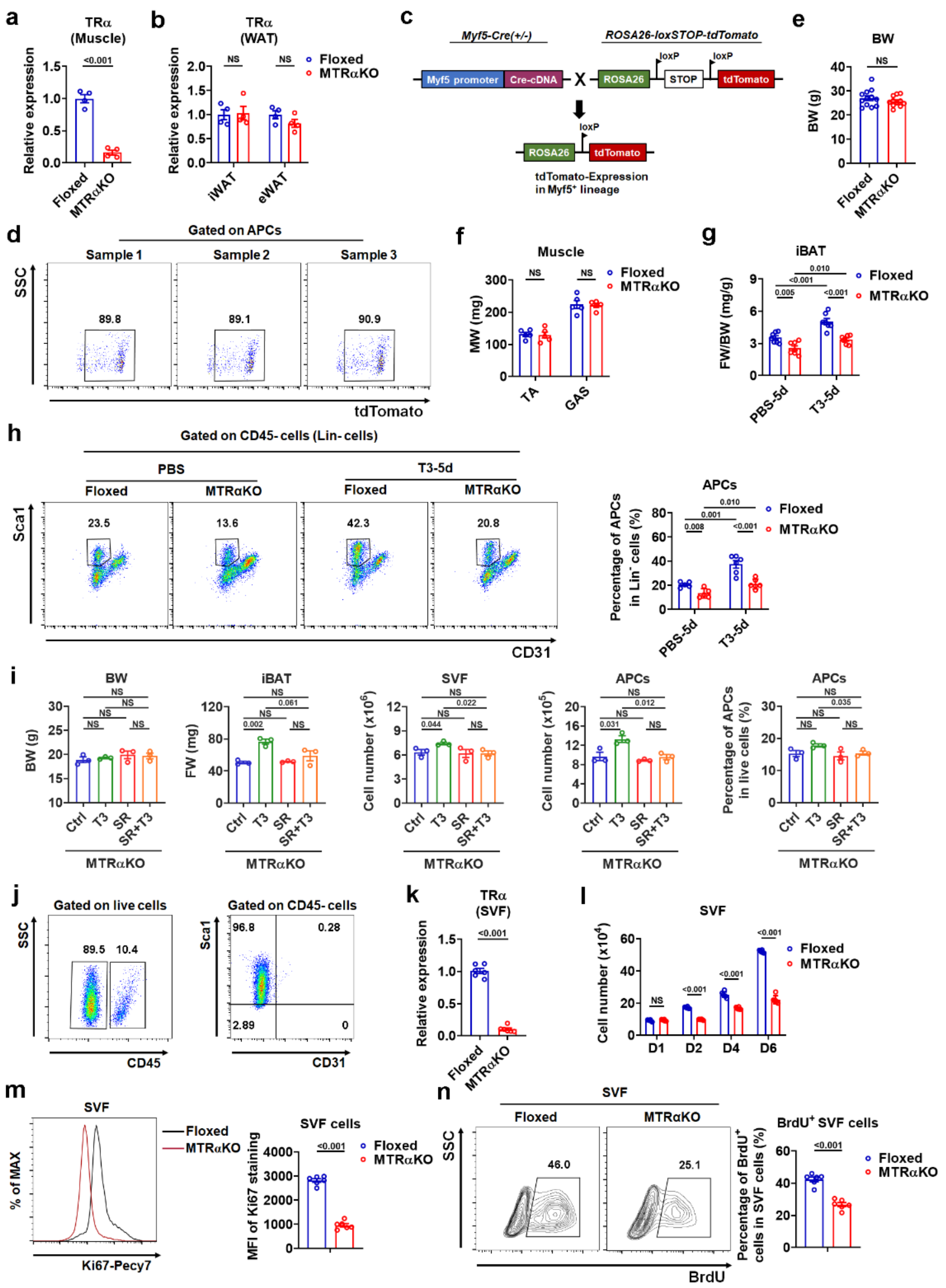

Supplementary Figure 4. Deletion of TRα with Myf5-Cre blunts T3 effect on iBAT hyperplasia.

(a and b) Relative TRα mRNA levels in muscle (a) and iWAT and eWAT (b) of Floxed and MTRαKO mice (n = 4). (c and d) Breeding scheme for generating ROSA26-tdTomato reporter mice expressing Cre recombinase under the control of Myf5-promoter (c), and the expression of tdTomato in APCs isolated from the iBAT of these mice (d). (e) BW of Floxed (n = 12) and MTRαKO (n = 11) mice. (f) Muscle weight (MW) of TA and GAS of Floxed and MTRαKO mice (n = 5). (g) FW/BW of iBAT of Floxed and MTRαKO mice with daily PBS or 0.5X standard dose of T3 injection for 5 d (n = 7). (h) FACS plot of APCs (left) and percentage of APCs in Lin$^-$ cells (right) from the iBAT of Floxed and MTRαKO mice with daily PBS (n = 5) or 0.5X standard dose of T3 (n = 6) injection for 5 d. (i) BW, FW of iBAT, number of SVF cells and APCs in iBAT per mouse and percentage of APCs in live SVF cells of iBAT from MTRαKO mice with daily SR59230A (SR) or T3 injection for 5 d (n = 3). (j) Flow cytometric analysis of cultured SVF cells. (k-n) Relative mRNA TRα levels (k, n = 6), cell number (l, n = 6), Ki67 expression (m, n =

6), and percentage of $BrdU^+$ cells (n, n = 6) in cultured SVF cells from the iBAT of Floxed and MTRαKO mice. Means ± SEM are shown. *P* values were calculated by two-tailed unpaired Student's *t* test for a-b, e-i, k-n. NS, not significant. Source data are provided as a Source Data file.

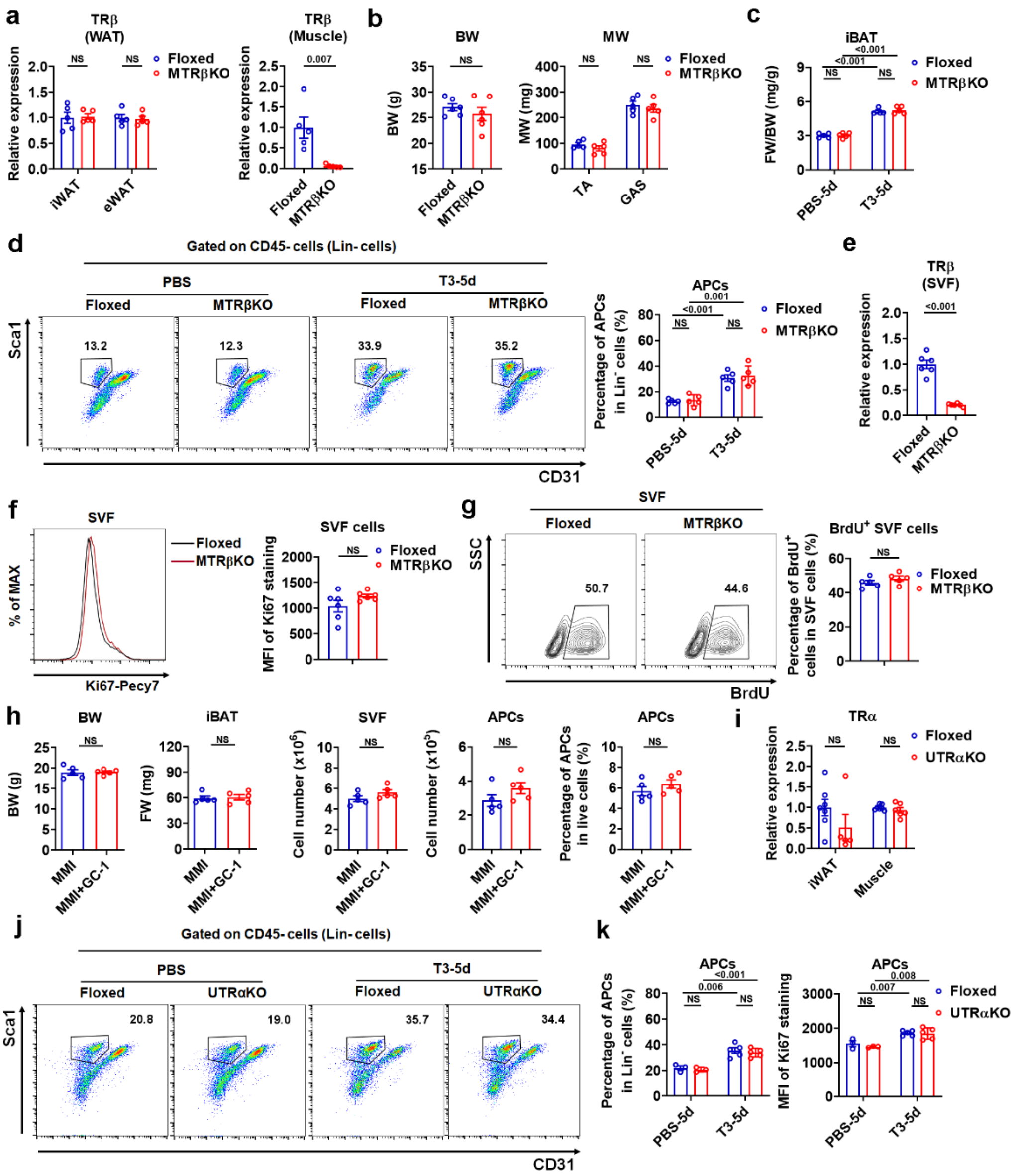

Supplementary Figure 5. Deletion of TRβ by Myf5-cre or TRα by UCP1-Cre barely affects APC proliferation in iBAT.

(a) Relative TRβ mRNA levels in iWAT and eWAT (left) and muscle (right) of Floxed and MTRβKO mice (n = 5). (b) BW (n = 6) and MW (n = 5) of Floxed and MTRβKO mice. (c and d) FW/BW of iBAT from Floxed and MTRβKO mice with daily PBS or 0.5X standard dose of T3 injection for 5 d (c, n = 5). FACS plot of APCs in iBAT (d, left) and percentage of APCs in Lin⁻ cells from iBAT (d, right, n = 5). (e-g) Relative TRβ mRNA levels in cultured SVF cells (e), flow cytometry histograms and quantification of Ki67 expression (f) and BrdU incorporation analysis of cultured SVF cells (g) from iBAT of Floxed and MTRβKO mice (n = 6). (h) BW, FW of iBAT, number of SVF and APCs in iBAT per mouse, and percentage of APCs in live SVF cells of iBAT from MMI mice and MMI mice with daily GC-1 injection for 5 d (n = 5). (i) Relative TRα mRNA levels in iWAT and muscle of Floxed (n = 7) and UTRαKO mice (n = 5 for iWAT, n = 6 for muscle). (j and k) FACS plot of APCs in iBAT (j), percentage of APCs in Lin⁻ cells from iBAT (k, left), and MFI of Ki67 in APCs from iBAT (k, right) of Floxed (n = 3) and UTRαKO (n = 5) mice with daily PBS or 0.5X standard dose of T3 injection for 5 d. Means ± SEM are shown. *P* values were calculated by two-tailed unpaired Student's *t* test for a-i, and k. NS, not significant. Source data are provided as a Source Data file.

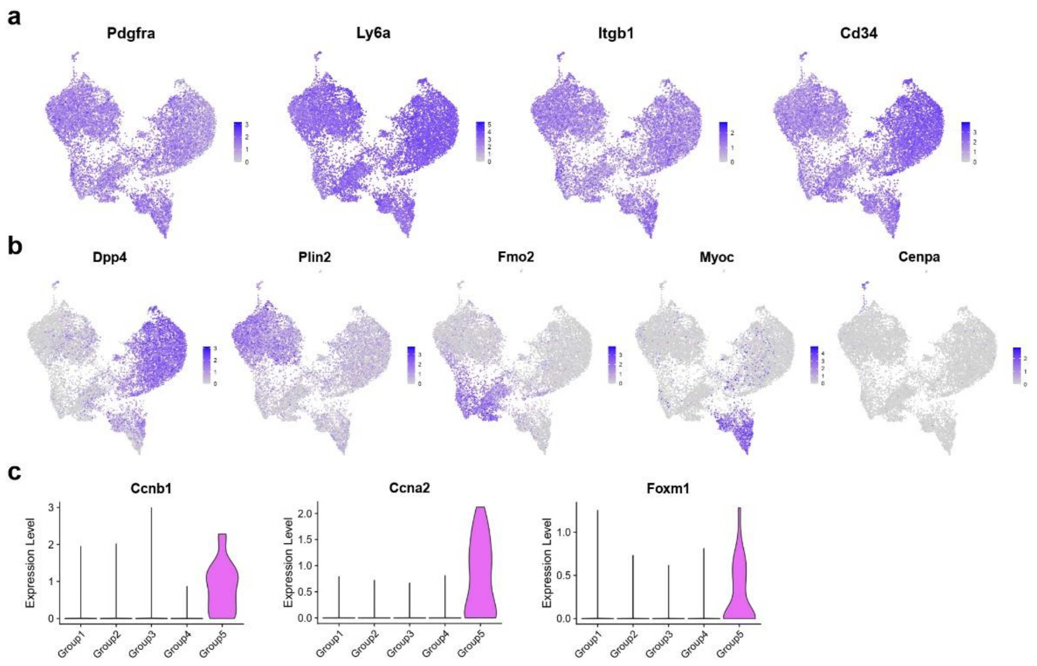

Supplementary Figure 6. scRNA-seq analysis reveals cellular heterogeneity of APCs in iBAT.

(a) Feature plots of expression levels and distribution for mesenchymal stem cell markers in APCs of iBAT. (b) Feature plots of expression levels and distribution for selected group-specific genes in APCs of iBAT. (c) Violin plots showing the expression levels of *Ccnb1*, *Ccna2*, and *Foxm1* in APCs of iBAT.

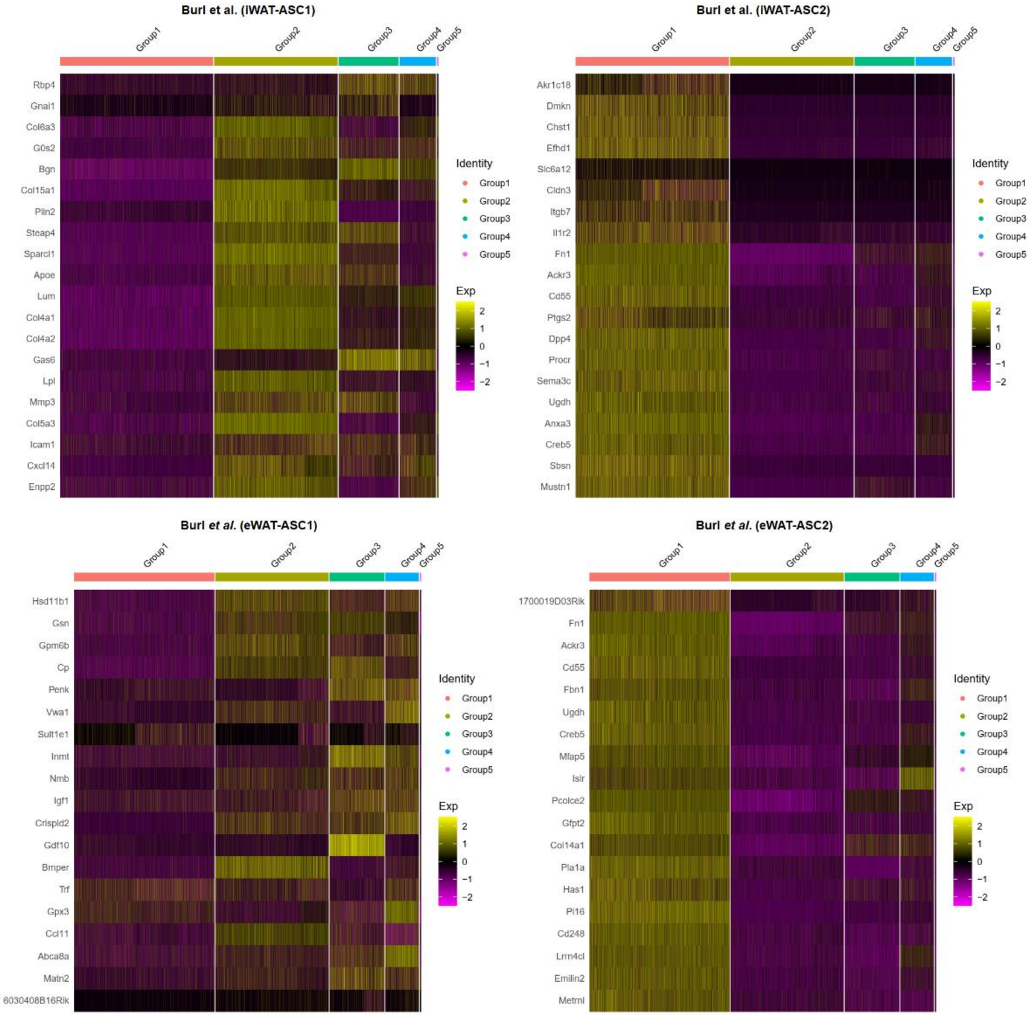

Supplementary Figure 7. Comparison of the data reported by Burl et al. with ours.

The top 20 cluster-defining genes of iWAT for their ASC1 (top, left) and ASC2 (top, right) populations are overlaid on our iBAT scRNA-seq scaled data (only the highly variable genes in our data are shown, the same below). The top 20 cluster-defining genes of eWAT for their ASC1 (bottom, left) and ASC2 (bottpm, right) populations are overlaid on our iBAT scRNA-seq scaled data.

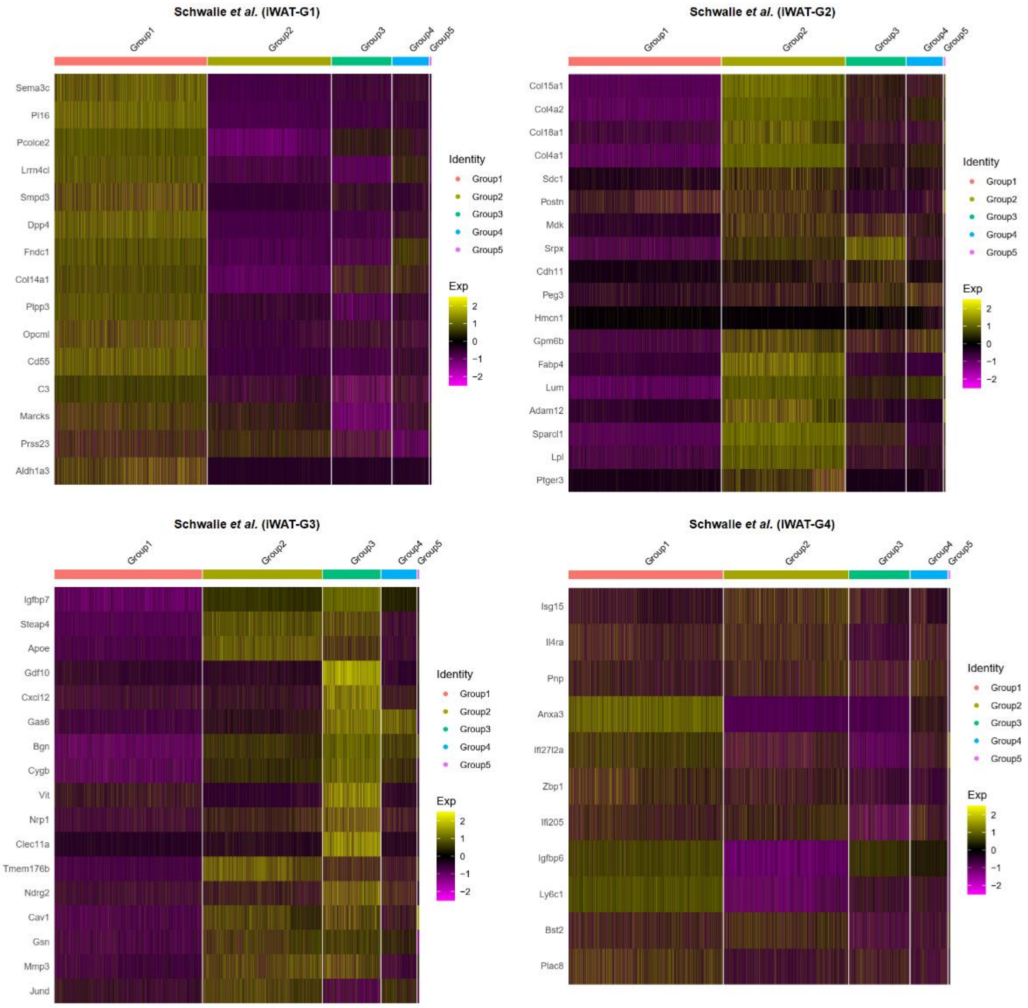

Supplementary Figure 8. Comparison of the data reported by Schwalie et al. with ours.

The top 20 cluster-defining genes for their G1 (top left), G2 (top right) , G3 (bottom left) and G4 (bottom right) populations are overlaid on our iBAT scRNA-seq scaled data.

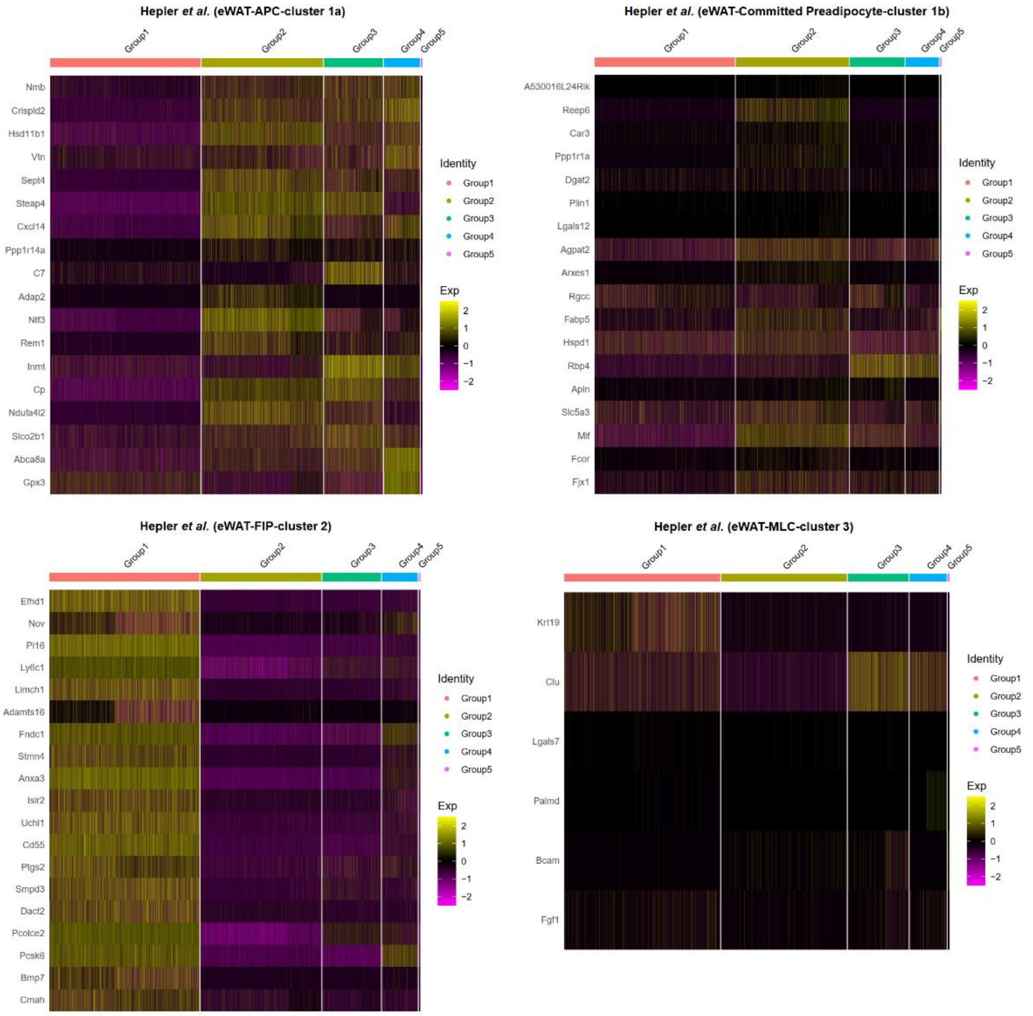

Supplementary Figure 9. Comparison of the data reported by Hepler et al. with ours.

The top 20 cluster-defining genes for their cluster1a (top left), cluster1b (top right), cluster 2 (bottom left) and cluster 3 (bottom right) populations are overlaid on our iBAT scRNA-seq scaled data.

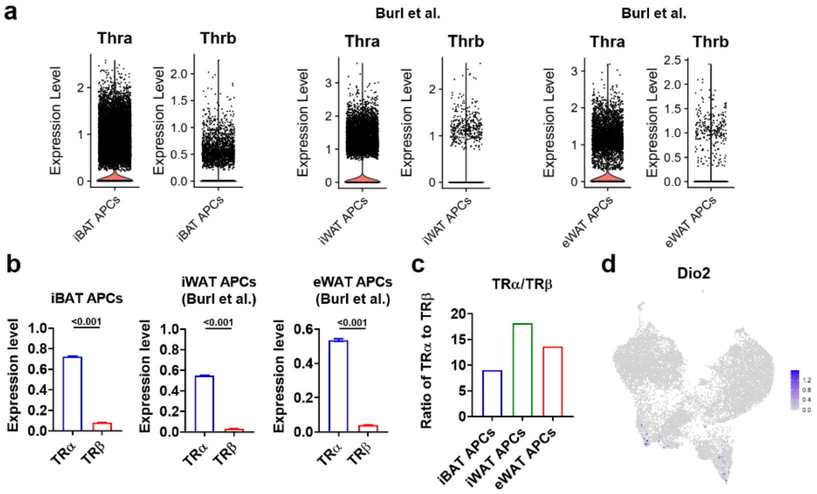

Supplementary Figure 10. Expression levels of TRα and TRβ in APCs of different types of adipose tissue, and Dio2 in APCs of iBAT.

(a-b) Dotted violin plots (a) and histograms (b) showing the expression levels of *Thra* (TRα), *Thrb* (TRβ) in APCs of iBAT (n = 12,715), iWAT (ASC1, ASC2 and Prolif. from iWAT reported by Burl et al.; n = 14,810) and eWAT (ASC 1, ASC 2, Diff. ASC and Pro. ASC from eWAT reported by Burl et al.; n = 7,259). (c) Ratio of TRα expression levels to TRβ expression levels in APCs of iBAT, iWAT and eWAT calculated by using the average expression levels of TRα and TRβ in panel b. (d) Feature plot showing the expression levels and distribution of *Dio2*. Means ± SEM are shown. *P* values were calculated by two-tailed unpaired Student's *t* test for b. Source data are provided as a Source Data file.

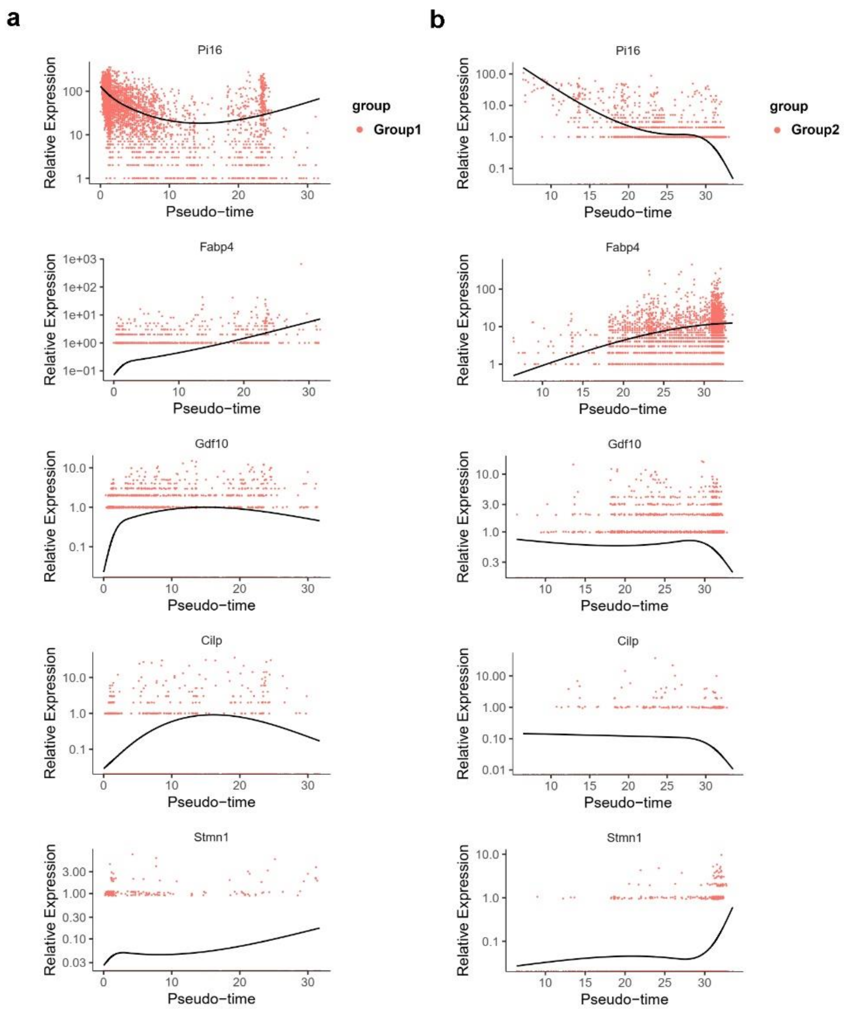

Supplementary Figure 11. Pseudotime developmental trajectories of Group 1 and 2 cells in iBAT.

(a) Pseudotime kinetics of representative differentially expressed genes (DEGs) between different groups along trajectories, only showing Group 1 cells. (b) Pseudotime kinetics of representative DEGs between different groups along trajectories, only showing Group 2 cells.

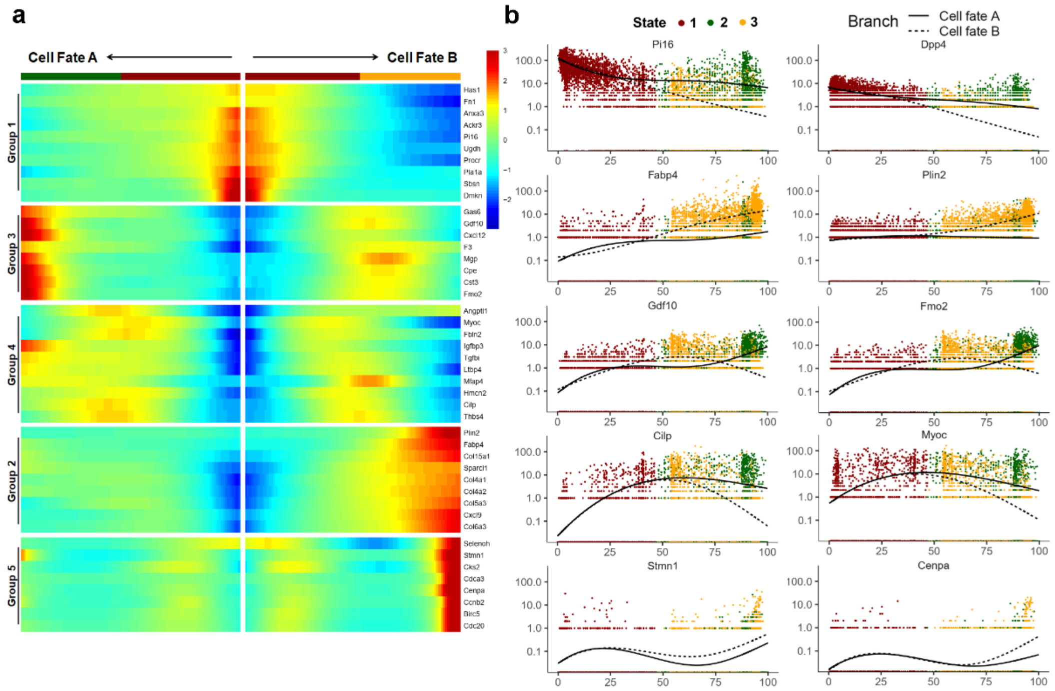

Supplementary Figure 12. Pseudotime developmental trajectories of APCs with different fates in iBAT.

(a) Heatmap showing the expression of DEGs between different groups in a branch-dependent manner. (b) Pseudotime kinetics of representative DEGs (*Pi16*, p = 0; *Dpp4*, p = 0; *Fabp4*, p = 0, *Plin2*, p = 0; *Gdf10*, p = 0; *Fmo2*, p = 0; *Cilp*, p = 0; *Myoc*, p = 0; *Stmn1*, p = 2.35E-23; *Cenpa*, p = 2.77E-22) between different groups from the root of trajectory to Fate A (solid line) and to Fate B (dashed line). The p values calculated by likelihood ratio test (two-tailed) reflected all the genes shown in panel d differ between the two cell fates.

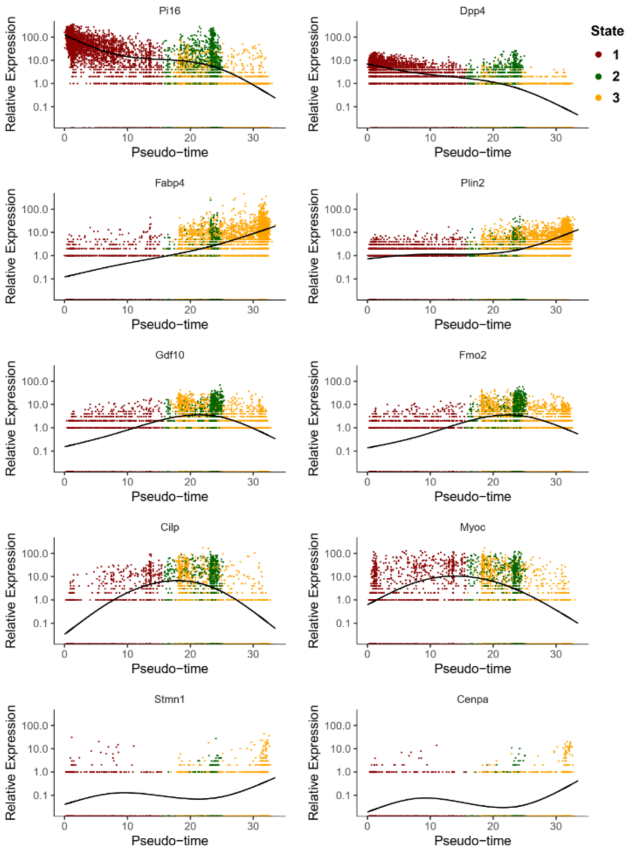

Supplementary Figure 13. Pseudotime developmental trajectories of APCs in different states in iBAT. Pseudotime kinetics of representative DEGs between different groups along trajectories.

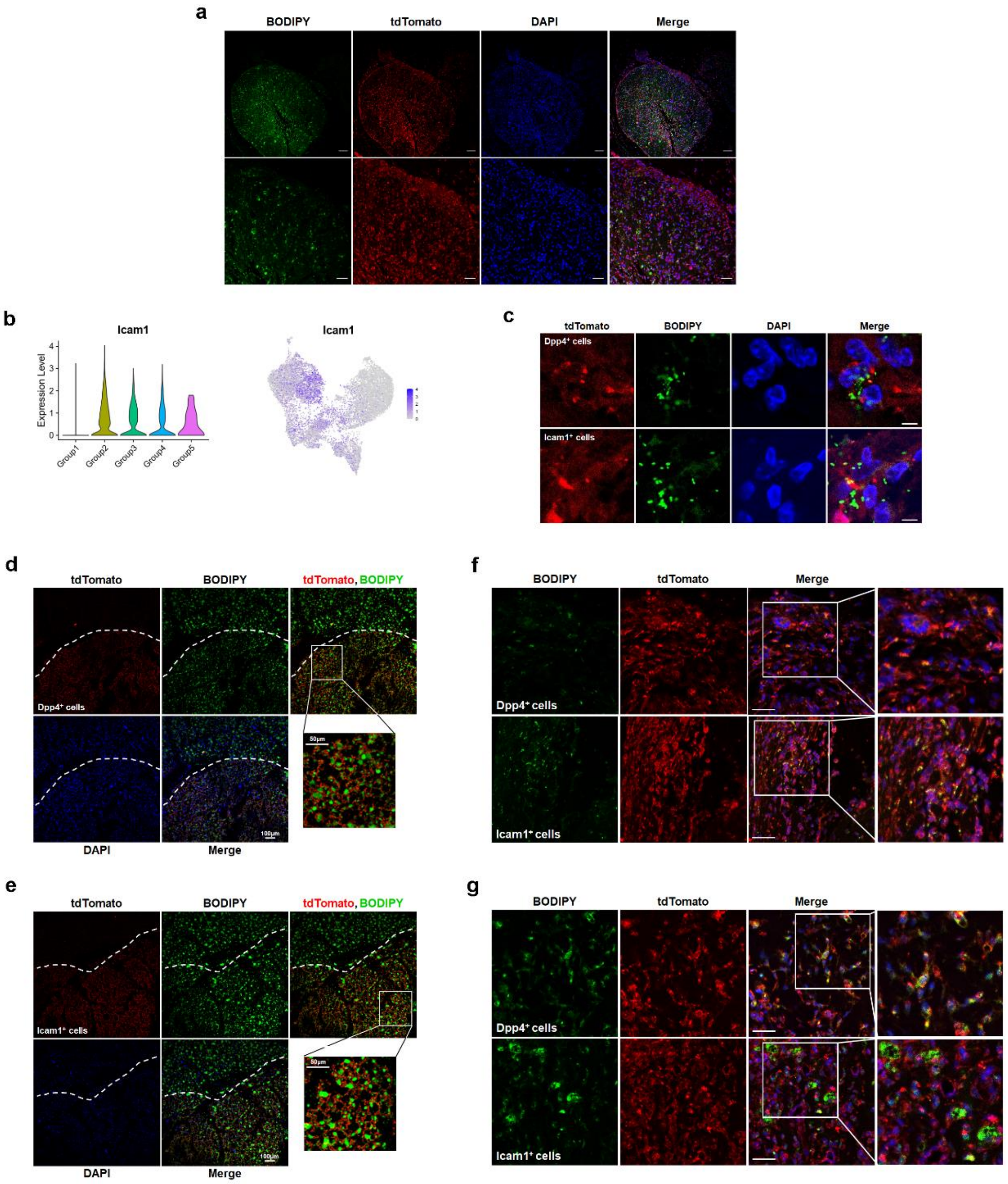

Supplementary Figure 14. *In vivo* adipogenic differentiation of progenitors.

(a) Immunofluorescence staining of APC-derived implants with BODIPY (green), tdTomato (red), and DAPI (blue) 14 days after transplantation. Scale bar, 100 μm (upper panels) or 50 μm (lower panels). (b) Violin plot (left) and feature plot (right) showing the expression levels and distribution of *Icam1*. (c) Immunofluorescence staining of tdTomato$^+$Dpp4$^+$ (upper panels) or tdTomato$^+$Icam1$^+$ (lower panels) APC-derived implants with BODIPY (green), tdTomato (red), and DAPI (blue) at 45 days posttransplantation. Scale bar, 10 μm. (d and e) Immunofluorescence staining of tdTomato$^+$Dpp4$^+$ (d) or tdTomato$^+$Icam1$^+$ (e) APC-derived implants with BODIPY (green), tdTomato (red), and DAPI (blue). One week after transplantation, mice were exposed to cold for 3 weeks. White dashed line indicates the boundary between implants and iBAT pad. Scale bar, 100 or 50 μm as indicated. (f and g) Immunofluorescence staining of Dpp4$^+$ (upper panels) or Icam1$^+$ (lower panels) APC-derived implants with BODIPY (green), tdTomato (red), and DAPI (blue) at 20 days (f) or 30 days (g) posttransplantation. Scale bar, 50 μm. 5×10^5 (panel d and e) APCs or 1×10^6 APCs (panel a, c, f, and g) were used for transplantation. The thickness of

tissue sections is 12 μm for panel d and e, and 10 μm for panel a, c, f, and g.

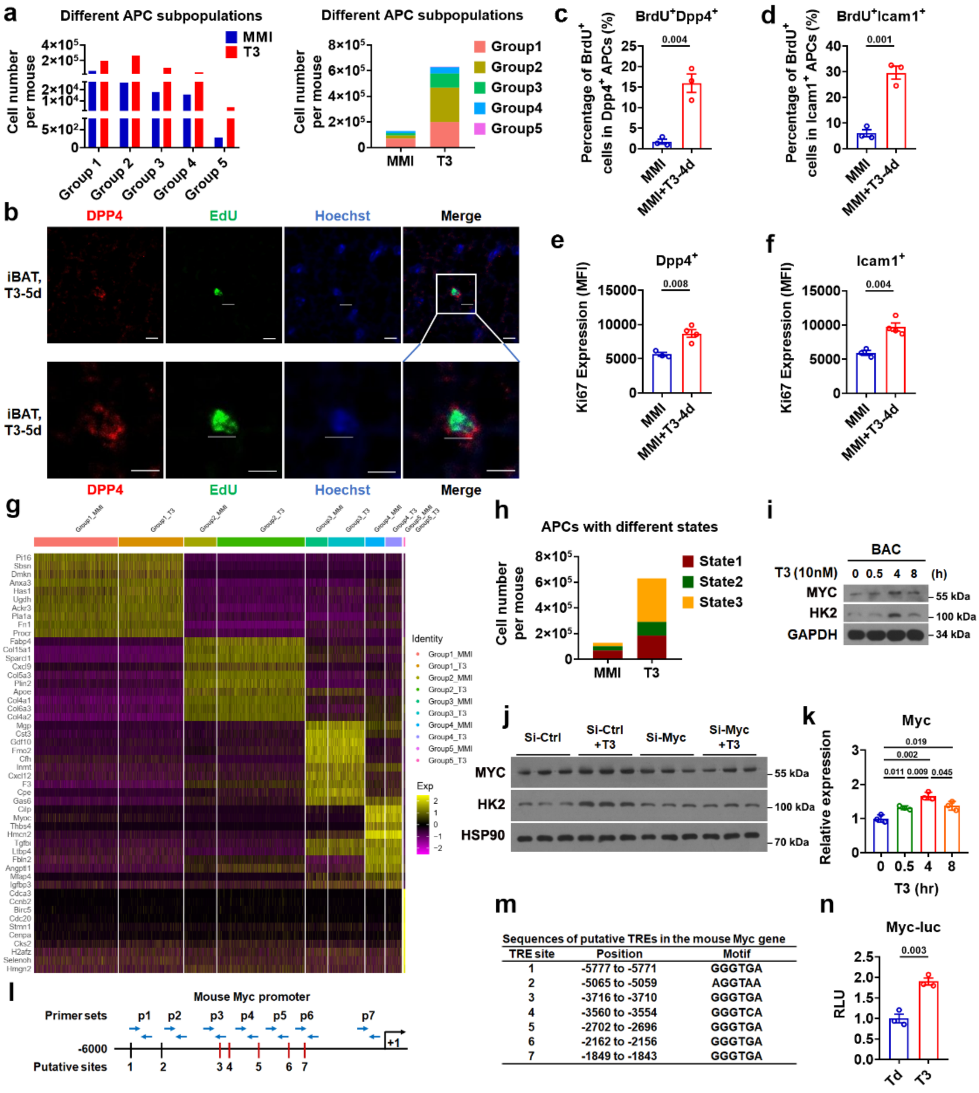

Supplementary Figure 15. T3 promotes cell state transition and cell cycle progression and regulates *Myc* transcription.

(a) Cell number of APCs from distinct groups in MMI group or T3 group per mouse. Data are plotted in a grouped graph (left) or in a parts-of-whole graph (right). (b) Immunofluorescence staining of Dpp4 and Hoechst with EdU labeling on iBAT sections from MMI mice with daily T3 injection at 0.5X standard dose for 5 d. Scale bar, 5 µm. (c and d) Percentage of $BrdU^+$ cells in $Dpp4^+$ APCs (c) and $Icam1^+$ APCs (d), respectively, in the iBAT of MMI mice and MMI mice with daily T3 injection at 0.5X standard dose for 4 d (n = 3). (e and f) MFI of Ki67 staining in $Dpp4^+$ APCs (e) and $Icam1^+$ APCs (f), respectively, in the iBAT of MMI mice (n = 3) and MMI mice (n = 4) with daily T3 injection at 0.5X standard dose for 4 d. (g) Heatmap showing scaled expression of DEGs between Group 1-5 (as shown in Fig. 5d) in MMI group and T3 group. (h) Cell number of APCs from distinct states in MMI group or T3 group per mouse. (i) Western blot analysis of Myc and Hk2 in BAC with T3 treatment for indicated time. (j) Western blot analysis of Myc and Hk2 in *Myc* siRNA-transfected BAC after T3 treatment. (k) qRT-PCR analysis of *Myc* in BAC with T3 treatment for indicated time (n = 3). (l) Putative sites of TREs and primer sets at the mouse *Myc* promoter. (m) Sequences of putative TREs in the mouse *Myc* promoter region. (n) Luciferase reporter assay to analyze the mouse *Myc* promoter activity in HEK293T cells (n = 3). Means ± SEM are shown. P values were calculated by two-tailed unpaired Student's t test for c-f, k, and n. Source data are provided as a Source Data file.

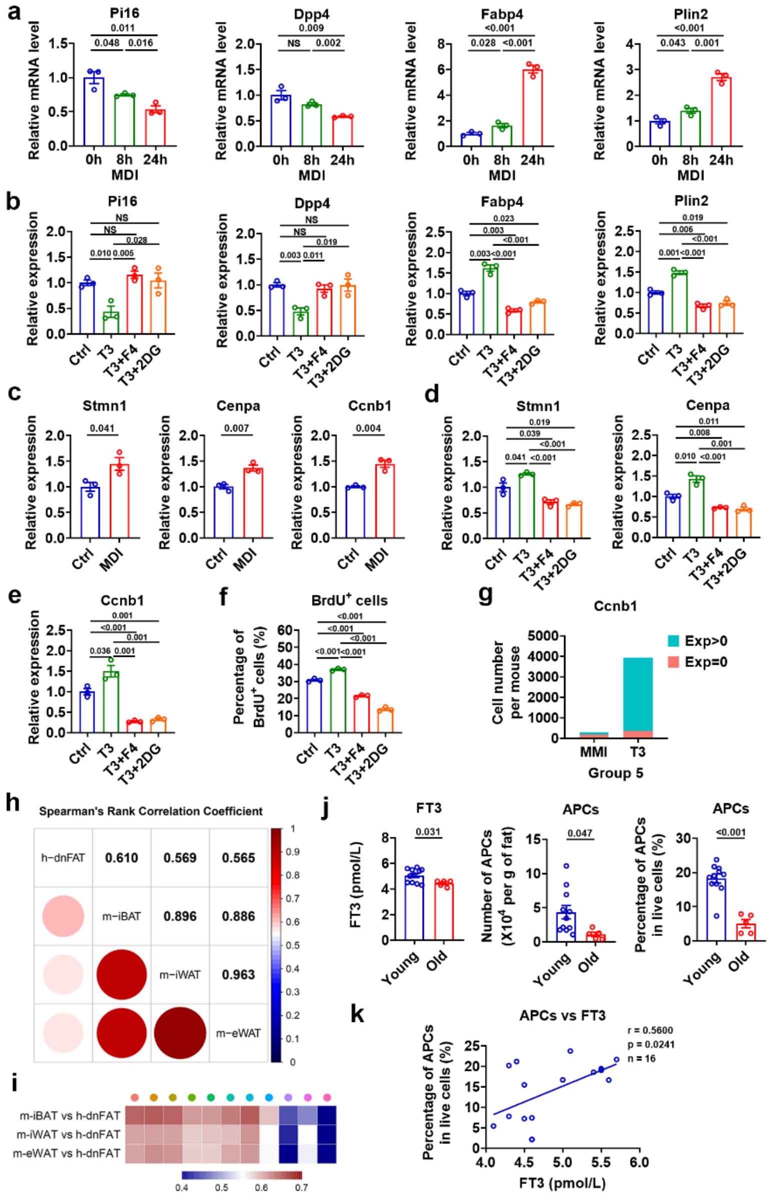

Supplementary Figure 16. Inhibition of Myc or glycolysis attenuates the T3 effect on cell state transition and cell cycle progression.

(a) qRT-PCR analysis of selected Group 1-specific genes and Group 2-specific genes in Dpp4^{+} cells after minimal adipogenic induction for indicated time (n = 3). MDI refers to the Mixture of the Differentiation Inducers. (b) qRT-PCR analysis of selected Group 1-specific genes and Group 2-specific genes in Dpp4^{+} cells exposed to minimal adipogenic conditions and treated with T3, 2DG, or F4-10058 (F4) as indicated (n = 3). (c) qRT-PCR analysis of Group 5 markers (*Stmn1* and *Cenpa*) and G2/M phases marker (*Ccnb1*) in Dpp4^{+} cells after minimal adipogenic induction for 24 h (n = 3). MDI refers to the Mixture of the Differentiation Inducers. (d and e) qRT-PCR analysis of Group 5 markers (*Stmn1* and *Cenpa*) (d) and G2/M phases marker (*Ccnb1*) (e) in Dpp4^{+} cells exposed to minimal adipogenic conditions and treated with T3, 2DG, or F4 as indicated (n = 3). (f) BrdU incorporation analysis of Dpp4^{+} cells exposed to minimal adipogenic conditions and treated with T3, 2DG, or F4 as indicated (n = 3). (g) Comparison of the number of *Ccnb1* positive cells per mouse in Group 5 cells between MMI group and T3 group. (h) Corrplot showing spearman rank correlation of global expression signature between preadipocytes from human deep-neck fat (h-dnFAT), APCs from mouse iBAT (m-iBAT), APCs from mouse iWAT (m-iWAT) and APCs from mouse eWAT

(m-eWAT). (i) Heatmap showing cross-species pairwise correlation between the matched subpopulations in preadipocytes or APCs from h-dnFAT, m-iBAT, m-iWAT, or m-eWAT. Colored circles represent matched subpopulations. (j) Free T3 levels, number of APCs per g of fat, and percentage of APCs in corresponding live SVF cells in young (ages < 65, n = 11) and old (ages > 65, n = 5) human subjects. (k) Correlation between free T3 level and percentage of APCs in live SVF cells of brown fat (neck fat) from human subjects (n = 16). Means ± SEM are shown. *P* values were calculated by two-tailed unpaired Student's *t* test for a-f, j, and was analyzed by Pearson correlation with two-tailed *t* test for k. NS, not significant. Source data are provided as a Source Data file.

Supplementary Table 1. Results of two-way ANOVA for data in Fig. 3g with treatment and genotype as main factors.

Source	Type III Sum of Squares	df	Mean Square	F	P value	% of total variation
Interaction	51.63	1	51.63	4.526	0.0475	6.354
Treatment (PBS/T3)	244.7	1	244.7	21.45	0.0002	30.12
Genotype (Floxed/MTRαKO)	285.8	1	285.8	25.05	<0.0001	35.17
Residual	205.3	18	11.41			

Supplementary Table 2. Results of two-way ANOVA for data in Fig. 3h with treatment and genotype as main factors.

Source	Type III Sum of Squares	df	Mean Square	F	P value	% of total variation
Interaction	66.59	1	66.59	4.397	0.0504	7.319
Treatment (PBS/T3)	151.8	1	151.8	10.02	0.0053	16.68
Genotype (Floxed/MTRαKO)	385.7	1	385.7	25.47	0.0001	42.4
Residual	272.6	18	15.14			

Supplementary Table 3. Results of two-way ANOVA for data in Fig. 3i with treatment and genotype as main factors.

Source	Type III Sum of Squares	df	Mean Square	F	P value	% of total variation
Interaction	182368	1	182368	4.92	0.0397	5.424
Treatment (PBS/T3)	1451649	1	1451649	39.16	0.0001	43.17
Genotype (Floxed/MTRαKO)	974362	1	974362	26.28	0.0001	28.98
Residual	667248	18	37069			

Supplementary Note

Supplementary Note 1

(From Results: T3 boosts the thermogenic capacity by promoting iBAT expansion)

H&E staining of iBAT sections revealed that the lipid droplets maintain their typical multilocular appearance after either short-term or long-term T3 treatment (Supplementary Fig. 1a). Consistent with the roles of T3 in promoting lipid utilization in brown adipocytes and driving systemic fat mobilization and lipogenesis in brown adipocytes [1, 2], the size of lipid droplet was slightly reduced after short-term T3 treatment, while lipid droplets became larger after long-term T3 treatment.

Dose- and time-dependent effects of T3 on iBAT size, weight, and the number of SVF cells from iBAT could be observed in MMI mice, respectively (Fig. 1h-k and Supplementary Fig. 1h). These results further indicate that T3 can increase the number of SVF cells in iBAT depot, which might contribute to the hyperplastic growth of iBAT.

Supplementary Note 2

(From Results: T3 expands the adipocyte progenitor population in iBAT depot)

To test whether the effect of i.p. injection of T3 on APC proliferation in the iBAT requires adrenergic activity, we performed experiments at thermoneutral temperature (30°C) or by using antagonists for adrenergic receptor (AR). We found that T3 treatment could increase the iBAT mass, the number of APCs, and the percentage of APCs in the iBAT of euthyroid mice under a thermoneutral condition in which thermal stress was eliminated (Supplementary Fig. 3a). We then compared the effect of i.p. injection of T3 on APC proliferation in iBAT depot between MMI mice housed at 30°C and room temperature (RT), a sub-thermoneutral temperature. Consistently, the T3 effect on the number and percentage of APCs were observed either at RT or 30°C (Supplementary Fig. 3b). We noticed a small but significant reduction in the number and percentage of APCs in T3-treated MMI mice housed at 30°C as compared to those T3-treated MMI mice housed at RT (Supplementary Fig. 3b). Given that cold-induced thermogenesis is nullified and sympathetic drive to BAT is minimal at thermoneutrality, these results not only indicate that the cold-induced sympathetic activation might be dispensable for the regulation of APC proliferation by T3, but also suggest that the sympathetic activation might be able to potentiate the T3 action and might be required for T3 to achieve its maximal effect on APC proliferation in iBAT.

Accordingly, the effect of i.p. injection of T3 on APC proliferation in iBAT could be observed after the treatment with either SR59230A (SR), a β3-AR antagonist, or

metoprolol (Met), a β1-AR antagonist (Supplementary Fig. 3c,d). Decreases could also be detected in the number and percentage of APCs in the iBAT of T3-treated MMI mice with either SR or Met treatment as compared to those in the iBAT of T3-treated MMI mice without SR or Met treatment (Supplementary Fig. 3c,d). These results suggest that, although either the β3 or β1-AR-mediated sympathetic activation might be dispensable for the regulation of APC proliferation by T3, both of them might be capable of potentiating the T3 action and might be required for T3 to achieve its maximal effect on APC proliferation in iBAT. Given that β1-AR and β3-AR have differential expression pattern and distinct functions in adipocyte progenitors and mature adipocytes, respectively [3, 4, 5], these results imply that both β1 and β3 adrenergic signaling are involved in the regulation of APC proliferation by T3 but the underlying mechanisms differ. As T3 regulates APC proliferation in a cell-autonomous manner (Fig. 2l and Supplementary Fig. 2i) and either β3 or β1-AR-mediated sympathetic activation is not required for the regulation of APC proliferation by T3, we speculate that either β1 or β3 adrenergic signaling contributes to the profound effect of T3 on APC proliferation but both of them are not indispensable in iBAT.

To test whether the central action of T3 also contributed to the increased APC proliferation in iBAT after i.p. injection of T3, we performed intracerebroventricular (ICV) injection of T3. Consisted with previous reports [6, 7, 8], ICV injection of T3 for 5 days increased the size of brown adipocytes in iBAT and decreased the size of white adipocytes in iWAT (Supplementary Fig. 3e). Moreover, increased UCP1 and tyrosine hydroxylase protein expression could be observed in both iBAT and iWAT after ICV injection of T3 (Supplementary Fig. 3f). Interestingly, we found that the number of APCs in iBAT was not significantly altered, while a decrease in the percentage of APCs was detected in iBAT, probably due to an increase in the percentage of endothelial cells (ECs) after ICV injection of T3 (Supplementary Fig. 3g). These results indicate that the central action of T3 might predominantly contribute to the regulation of thermogenesis in iBAT depot through increasing the UCP1 expression in mature brown adipocytes, it might not contribute to the T3-induced APC proliferation in iBAT, or it might not participate directly in the regulation of APC proliferation in iBAT by circulating T3 or local T3 generated in mature brown adipocytes via type 2 deiodinase (Dio2), which catalyzes the conversion of prohormone thyroxine (T4) to the bioactive TH (T3).

To avoid systemic effects that may complicate interpretation, we employed an approach developed recently by using rolling microneedles for local delivery of T3 to iBAT region. The tissue-specific T3 delivery was evaluated in TH action indicator (THAI) mice developed previously, which harbor a TH-responsive luciferase reporting

system [9]. We found that local injection of T3 through microneedles into the iBAT region for 5 days only increased the luciferase activity in iBAT but not in other T3 target tissues, suggesting that this rolling microneedle-based approach could achieve an iBAT-specific T3 administration (Supplementary Fig. 3h). In line with the data obtained in mice with i.p. injection of T3, iBAT-specific T3 treatment increased the iBAT mass and the number of APCs in the iBAT depot (Supplementary Fig. 3i). Although we could not exclude the possibility that other factors or other tissues might also be involved in the regulation of iBAT hyperplasia by the elevation of circulating T3 levels after i.p. injection of T3, these results together with our above in vivo and in vitro data suggest that T3 is capable of targeting APCs resided in iBAT directly and promoting APC proliferation in a cell-autonomous manner.

Supplementary Note 3

(From Results: Deletion of TRα with Myf5-Cre attenuates the T3 effect on hyperplasia of iBAT)

We crossed TRβ flox/flox mice (TRβFloxed mice) that we developed recently with Myf5-Cre mice to generate mice without TRβ in Myf5-lineage (MTRβKO mice) (Fig. 4a). As expected, the mRNA expression of TRβ was greatly decreased in iBAT and skeletal muscle, but not altered in WATs (Fig. 4b and Supplementary Fig. 5a). Similar to MTRαKO mice, MTRβKO mice exhibited normal body weight and muscle weight (Supplementary Fig. 5b). However, in contrast to MTRαKO mice, the iBAT size and weight were not altered in MTRβKO mice compare to TRβFloxed mice, while loss of TRβ in Myf5-lineage had no effect on T3-induced enlargement of iBAT (Fig. 4c and Supplementary Fig. 5c). Moreover, the number and percentage of APCs were not altered in the iBAT of MTRβKO mice compared to TRβFloxed mice (Fig. 4d,e and Supplementary Fig. 5d). Furthermore, loss of TRβ in Myf5-lineage had no effect on T3-induced increases in either the number or the percentage of APCs (Fig. 4d,e and Supplementary Fig. 5d). Accordingly, no difference was observed in Ki67 staining intensity of APCs between MTRβKO and TRβFloxed mice either with or without i.p. injection of T3 (Fig. 4f). Analysis of in vivo BrdU labeling did not reveal any difference in the percentage of $BrdU^+$ APCs between MTRβKO and TRβFloxed mice after i.p. injection of T3 (Fig. 4g). Consistently, the in vitro data obtained from the cultured APC-enriched SVF cells derived from the iBAT of MTRβKO and TRβFloxed mice showed that loss of MTRβ had no effect on either the Ki67 staining intensity of SVF cells or the percentage of $BrdU^+$ SVF cells (Supplementary Fig. 5e-g). Additionally, we treated MMI mice with i.p. injection of GC-1, a TRβ-selective agonist. In line with above data, GC-1 administration was not able to alter the number and percentage of APCs in iBAT

(Supplementary Fig. 5h). Collectively, these in vivo and in vitro results suggest that the TRβ in Myf5-lineage is dispensable for T3 induced-hyperplasia of iBAT.

As iBAT mainly originates from Myf5^{+} progenitors [10], MTRαKO mice also lost TRα in mature brown adipocytes due to Myf5-Cre-mediated recombination. To test whether TRα in mature brown adipocytes contributes to the T3 effect on APCs in vivo, we crossed TRαFloxed mice with UCP1-Cre mice to generate mice with mature brown adipocyte-specific TRα knockout (UTRαKO mice) (Fig. 4h). As expected, the mRNA expression of TRα was dramatically decreased in the iBAT of UTRαKO mice (Fig. 4i), while no changes were observed in the inguinal WAT (iWAT) and skeletal muscle (Supplementary Fig. 5i). Interestingly, the number and percentage of APCs, as well as the Ki67 staining intensity of APCs were not altered in the iBAT of UTRαKO mice (Fig. 4j,k and Supplementary Fig. 5j,k). Similar results were observed in UTRαKO mice after i.p. injection of T3 (Fig. 4j,k and Supplementary Fig. 5j,k). Additionally, the percentage of newly generated APCs was not altered in the iBAT of UTRαKO mice after i.p. injection of T3 (Fig. 4l). These results indicate that the TRα in brown progenitors but not the TRα in mature brown adipocytes predominantly mediates the T3 effect on the proliferative capacity of APCs in iBAT.

Supplementary Note 4

(From Results: ScRNA-seq analysis reveals the heterogeneity of APCs in iBAT depot)

The scRNA-seq of the APCs yielded data for 5,513 and 7,202 cells that passed the quality control filtering from MMI mice (referred to as MMI group, n = 30 mice pooled) and MMI mice treated with T3 for 4 days (referred to as T3 group, n = 8 mice pooled), respectively. We then combined all these cells (12,715 cells in total) from these two groups and performed unsupervised clustering using the Seurat R package. As expected, the canonical mesenchymal progenitor markers *Pdgfra*, *Ly6a* (*Sca1*), *Itgb1* (*Cd29*), and *Cd34*, were expressed in all subpopulations (Supplementary Fig. 6a).

Group 1 (cluster 0/3/6/8/9, accounting for ~40.9% of total APCs) cells expressed high levels of *Pi16* and *Dpp4*, but did not exhibit expression of adipocyte markers, indicating that cells in Group 1 are more primitive undifferentiated APCs. Group 2 (cluster 1/2, ~33.0%) cells were marked by high expression of *Fabp4* and *Plin2*, indicating that these cells are more committed preadipocytes. Group 3 (clusters 4/7, ~16.0%) and Group 4 (cluster 5, ~9.8%) cells exhibited high expression of *Gdf10* and *Fmo2* (Group 3) and high levels of *Cilp* and *Myoc* (Group 4), respectively. Group 5 (cluster 10, ~0.45%) cells expressed high levels of *Stmn1* and *Cenpa* (Fig. 5d-j,

Supplementary Fig. 6b and Supplementary Data 2).

The proliferative and adipogenic capacity of adipocyte progenitors is significantly affected by the location of the fat depot from which they were isolated and diverse conditions such as aging and obesity. Recently, a couple of studies have resolved the APC heterogeneity in mouse subcutaneous iWAT and visceral epigonadal WAT (eWAT), respectively. Despite differences in methodologies and/or mouse models, these studies in mouse stratified APCs into 2 to 4 main subpopulations. To compare the results of these different studies, top cluster-defining genes reported by Burl et al. [11], Schwalie et al. [12], and Hepler et al. [13] were overlaid on our mouse scRNA-seq data and group identities (Supplementary Fig. 7-9). We found that the group-defining genes for ASC2 cells (Burl et al.), both in eWAT and iWAT, overlap extensively with the genes defining our Group 1 population. Fibroinflammatory precursors (FIPs) (Hepler et al.) and G1 (Schwalie et al.) are also similar to our Group 1 cells. Our Group 2 cells, are more similar to the ASC1 cells (Burl et al.) of iWAT and G2 (Schwalie et al.) than ASC1 cells (Burl et al.) of eWAT and adipocyte precursor cells (APCs) (Hepler et al.) of eWAT. On the whole, our Group 1 cells corresponding to ASC2 cells (Burl et al.), fibroinflammatory precursors (FIPs, Hepler et al.) and G1 (Schwalie et al.), represent a highly conserved population among different fat depots, while our Group 2 cells in iBAT are more similar to corresponding cell population of iWAT than to those in eWAT. Thus, our study sheds light on how cellular APCs landscape differs among different fat depots and to what extent these differences reflect their physiological features.

Consistent with the data obtained by qPCR analysis (Fig. 3a), our scRNA-seq analysis revealed that the mRNA levels of TRα in APCs from iBAT were 9 times higher than those of TRβ in APCs from iBAT (Supplementary Fig. 10a-c), further suggesting that TRα is the major TR isoform in the APCs resided in the iBAT, which may play a more important role than TRβ in these APCs. Notably, the mRNA levels of TRα and TRβ are similar in adipocytes differentiated from iBAT-derived SVF, suggesting that both TRα and TRβ might be important for the function of mature adipocytes (Fig. 3a). Based on our findings here, we speculate that TRα might be more important for proliferation than TRβ in adipose progenitor cells (at least in APCs derived from $Myf5^+$ lineage), while both TRα and TRβ might be important for brown adipocyte maturation and function. We also analyzed the scRNA-seq data reported by Burl et al. [11] and found that the mRNA levels of TRα were 18 times and 14 times higher than those of TRβ in the APCs resided in iWAT and eWAT, respectively, suggesting that TRα might also be critically involved in the regulation of APCs resided in WAT (Supplementary Fig. 10a-c). Current evidence based on the studies using global TRβ KO mice and TRβ agonist

GC-1 suggests that TRβ plays a critical role in the browning of WAT [14, 15]. Given that the evidence showing the importance of TRα in beigeing is lacking, whether there is a different usage of the receptor for each thermogenic depot, and whether TRα and TRβ play a specific or redundant role in certain cell types requires further studies in the future, which may strategize future therapeutic interventions by targeting specific TR isoform in certain cell types.

Dio2 expression is enriched in mature brown adipocytes as compared to that in brown preadipocytes. It has been proposed that, upon thermogenic stress, the local production of T3 is increased in BAT due to the stimulated deiodination catalyzed by Dio2, and thus BAT can be a major source of circulating T3 [16, 17, 18]. In this study, we found that the abundance of *Dio2* was low in APCs (Supplementary Fig. 10d). Based on our findings, we speculate that cold-induced neural activation might increase the T3 generation in mature adipocytes via Dio2, leading to an elevation of local T3 levels, thereby promoting the APC proliferation within the iBAT depot.

Supplementary Note 5

(From Results: Pseudotemporal trajectory analysis delineates the hierarchy of APCs in iBAT)

To test our hypothesis, the expression of group markers along the pseudotime trajectory towards Cell Fate A and Cell Fate B, was assessed (Supplementary Fig. 12a,b, and Supplementary Fig. 13). The expression of Group 1 specific genes (*Pi16*, *Dpp4*, etc.) was gradually reduced along the progression of pseudotime with greater reduction observed in cells with Fate B than that in cells with Fate A. A gradual increase in the expression of Group 2 maker genes (*Fabp4*, *Plin2*, etc.) was predominantly observed along pseudotime in cells with Fate B but not in cells with Fate A. The expression of Group 3 specific genes (*Gdf10*, *Fmo2*, etc.) increased in the initial stage followed by a further increase in cells with Fate A or a decline in cells with Fate B in the late stage along pseudotime. The expression of Group 4 maker genes (*Cilp*, *Myoc*, etc.) peaked in the intermediate stage with a sharper decline in cells with Fate B than that in cells with Fate A. As *Pi16* and *Dpp4* expression reflects the characteristics of stem cells, while *Fabp4* and *Plin2* expression indicates adipogenic commitment, we proposed that Group 1 progenitors can adopt two fate trajectories along the pseudotime, giving rise to either the more committed Group 2 progenitors or the Group 3 cells. Notably, the expression of markers for Group 1 did not decline dramatically when stem-cell like cells adopted Fate A, suggesting that the cells would retain certain stem-like properties.

To validate the pseudotime trajectories of APCs in iBAT, we carried out in vivo cell

transplantation studies. We transplanted tdTomato-expressing APCs into the iBAT of mice. BODIPY staining of the implants after 2 weeks revealed lipid droplet-containing tdTomato^{+} cells (Supplementary Fig. 14a), suggesting that sort-purified APCs from the iBAT of tdTomato^{+} donor mice can differentiate in vivo. On the basis of the differential expression of cell-surface markers, Dpp4 (avg_logFC: 1.16, p_val_adj: < 1.0E-303) for Group 1 and Icam1 (avg_logFC: 0.38, p_val_adj: 2.30E-83) for Group 2, respectively (Fig. 5e and Supplementary Fig. 6b,14b), we transplanted tdTomato-expressing Dpp4^{+} APCs and Icam1^{+} APCs into the iBAT depot, respectively, for indicated period of time. BODIPY staining revealed lipid droplet-containing tdTomato^{+} cells in either Dpp4^{+} or Icam1^{+} APC-derived implants, suggesting that both sort-purified Dpp4^{+} and Icam1^{+} APCs can differentiate in vivo (Supplementary Fig. 14c).

Supplementary Note 6

(From Results: T3 promotes cell state transition and cell cycle progression in APCs of iBAT)

To test whether TR regulates *Myc* expression through direct binding to its promoter, we searched for T3 response elements (TREs) in the promoter region of mouse *Myc* gene (up to 6-kb upstream of the start site) and found multiple putative sites, including one site (site 3) conserved between mouse and human (Supplementary Fig. 15l). To further test which specific promoter regions might be responsible to the recruitment of TRα, we performed ChIP-PCR experiments (Supplementary Fig. 15l,m). Our results suggest that sites 3-7 but not sites 1 and 2 might be responsible to the recruitment of TRα (Fig. 8k). Accordingly, luciferase assay showed that T3 could stimulate the activity of the reporter containing 6-kb mouse *Myc* promoter fragment (Supplementary Fig. 15n). Collectively, these results indicate that *Myc* is a direct target gene of T3 and T3 may control glycolysis by direct regulating the transcription of *Myc* in the APCs of iBAT.

As metabolism has a central role in regulating cell state transition, we then tested whether increased glycolysis is required for the cell state transition of APCs induced by T3. In agreement with our notion that Group 1 cells are able to give rise to Group 2 cells, upon minimal adipogenic induction within 24 h, the mRNA expression of markers for Group 1 (*Pi16* and *Dpp4*) was decreased, while the mRNA levels of markers for Group 2 (*Fabp4* and *Plin2*) were elevated in cultured Dpp4^{+} APCs (Supplementary Fig. 16a). Interestingly, under the same condition, the mRNA levels of *Pi16* and *Dpp4* were lower and the mRNA levels of *Fabp4* and *Plin2* were higher, respectively, in the presence of T3 than those in the absence of T3 (Supplementary Fig. 16b).

We speculated that T3 may also play a role in mitotic progression along adipogenic trajectory. To test this hypothesis, we first examined the expression of Group 5 markers (*Stmn1* and *Cenpa*) and G2/M phases marker (*Ccnb1*) in cultured Dpp4$^+$ APCs after minimal adipogenic induction for 24 hours. We found that the mRNA levels of *Stmn1*, *Cenpa*, and *Ccnb1* were all increased, suggesting that adipogenic induction also promote mitotic commitment (Supplementary Fig. 16c). Interestingly, T3 treatment further enhanced the expression of these genes, while treatment of either 2DG or F4 attenuated the effect of T3 on the mRNA expression of these genes upon minimal adipogenic induction (Supplementary Fig. 16d,e). Accordingly, the BrdU incorporation was increased by T3 treatment, while either 2DG or F4 could attenuate the effect of T3 on the BrdU incorporation in cultured Dpp4$^+$ APCs after minimal adipogenic induction (Supplementary Fig. 16f).

Whether APCs resided in adult human BAT are similar to or different from APCs resided in murine beige fat and classic BAT has not been extensively studied. We compared our scRNA-seq data (APCs from mouse iBAT) with those reported by Burl et al. (ASC 1, ASC 2, and Prolif. from mouse iWAT; ASC 1, ASC 2, Pro. ASC and Diff. ASC from eWAT) [11], and Sun et al. (preadipocytes from human deep-neck fat) [19]. Correlation of global expression signature of preadipocytes from human deep-neck fat and APCs of three types of adipocytes from mice was measured by spearman rank correlation coefficient (CC). As expected, the highest CC was observed between mouse iWAT and mouse eWAT (CC: 0.963). Interestingly, the preadipocytes from human deep-neck fat were more closely related to APCs from mouse iBAT (CC: 0.61) than APCs from mouse iWAT (CC: 0.569) and mouse eWAT (CC: 0.565) (Supplementary Fig. 16h). We also used a recently developed method to determine the cross-species similarities based on scRNA-seq data, which requires cell types to be matched between species before correlations are calculated [20, 21]. We combined the four datasets to perform annotation and assigned 11 matched cell subpopulations between human and mouse. Spearman rank correlation of average expression levels in each matched subpopulation revealed a higher correlation between preadipocytes from human deep-neck fat and APCs from mouse iBAT (Supplementary Fig. 16i).

As hypothyroid mice had fewer APCs as compared to euthyroid mice (Supplementary Fig. 2g), we speculate that, at least at room temperature, normal TH levels are required for maintaining the stem cell pool and a euthyroid condition is critical for the normal physiology of BAT. As our analysis revealed a higher correlation between preadipocytes from human deep-neck fat and APCs from mouse iBAT (Supplementary Fig. 16h,i), we also assessed the physiopathological relevance of our

findings by using human samples. The correlation between the T3 level and the number of APCs in human neck brown fat (Supplementary Fig. 16k) also indicates that normal T3 level might be essential for maintaining the APC homeostasis in human brown fat.

Supplementary Discussion

(From Discussion)

Consistent with the results of scRNA-seq analysis, the percentage of most stem-like progenitors (Dpp4^{+}) was decreased rather than increased after long-term T3 treatment, while the percentage of more committed preadipocytes (Icam1^{+}) was increased correspondingly in the iBAT depot (Fig. 8c,d). Accordingly, T3 could reinforce the effect of minimal adipogenic induction, as evident by accelerated downregulation and upregulation of markers for Group 1 and Group 2, respectively (Supplementary Fig. 16b). On the other hand, our scRNA-seq analysis revealed a previously undescribed subpopulation (Group 5) in APCs of iBAT that is undergoing mitotic division (Fig. 5). After T3 treatment, the proportion of Group 5 cells increased greatly in the APCs of iBAT (Fig. 8b). Accordingly, addition of T3 could further enhance the expression of marker genes of Group 5 and G2/M phase marker in cultured adipocyte progenitors upon adipogenic induction (Supplementary Fig. 16d,e). Similar results could be observed in vivo after T3 treatment (Fig. 9b,h,i).

Supplementary References

1. Obregon MJ. Thyroid hormone and adipocyte differentiation. *Thyroid* **18**, 185-195 (2008).
2. Yeh WJ, Leahy P, Freake HC. Regulation of brown adipose tissue lipogenesis by thyroid hormone and the sympathetic nervous system. *Am J Physiol* **265**, E252-258 (1993).
3. Bronnikov G, Bengtsson T, Kramarova L, Golozoubova V, Cannon B, Nedergaard J. beta1 to beta3 switch in control of cyclic adenosine monophosphate during brown adipocyte development explains distinct beta-adrenoceptor subtype mediation of proliferation and differentiation. *Endocrinology* **140**, 4185-4197 (1999).
4. Lee YH, Petkova AP, Konkar AA, Granneman JG. Cellular origins of cold-induced brown adipocytes in adult mice. *FASEB J* **29**, 286-299 (2015).
5. Evans BA, Merlin J, Bengtsson T, Hutchinson DS. Adrenoceptors in white, brown, and brite adipocytes. *Br J Pharmacol* **176**, 2416-2432 (2019).
6. Martinez-Sanchez N*, et al.* Hypothalamic AMPK-ER Stress-JNK1 Axis Mediates the Central Actions of Thyroid Hormones on Energy Balance. *Cell Metab* **26**, 212-+ (2017).
7. Alvarez-Crespo M*, et al.* Essential role of UCP1 modulating the central effects of thyroid hormones on energy balance. *Mol Metab* **5**, 271-282 (2016).
8. Lopez M*, et al.* Hypothalamic AMPK and fatty acid metabolism mediate thyroid regulation of energy balance. *Nat Med* **16**, 1001-1008 (2010).
9. Mohacsik P*, et al.* A Transgenic Mouse Model for Detection of Tissue-Specific Thyroid Hormone Action. *Endocrinology* **159**, 1159-1171 (2018).
10. Seale P*, et al.* PRDM16 controls a brown fat/skeletal muscle switch. *Nature* **454**, 961-967 (2008).
11. Burl RB, Ramseyer VD, Rondini EA, Pique-Regi R, Lee YH, Granneman JG. Deconstructing Adipogenesis Induced by beta3-Adrenergic Receptor Activation with Single-Cell Expression Profiling. *Cell Metab* **28**, 300-309 e304 (2018).
12. Schwalie PC*, et al.* A stromal cell population that inhibits adipogenesis in mammalian fat depots. *Nature* **559**, 103-108 (2018).
13. Hepler C*, et al.* Identification of functionally distinct fibro-inflammatory and adipogenic stromal subpopulations in visceral adipose tissue of adult mice. *eLife* **7**, (2018).
14. Johann K*, et al.* Thyroid-Hormone-Induced Browning of White Adipose Tissue Does Not Contribute to Thermogenesis and Glucose Consumption. *Cell Rep* **27**, 3385-3400 e3383 (2019).
15. Lin JZ*, et al.* Pharmacological Activation of Thyroid Hormone Receptors Elicits a Functional Conversion of White to Brown Fat. *Cell Rep* **13**, 1528-1537 (2015).
16. Bianco AC, McAninch EA. The role of thyroid hormone and brown adipose tissue in energy homoeostasis. *Lancet Diabetes Endocrinol* **1**, 250-258 (2013).
17. Silva JE. The multiple contributions of thyroid hormone to heat production. *J Clin Invest* **108**, 35-37 (2001).
18. Silva JE. Thermogenic mechanisms and their hormonal regulation. *Physiol Rev* **86**, 435-464 (2006).
19. Sun W*, et al.* snRNA-seq reveals a subpopulation of adipocytes that regulates thermogenesis. *Nature* **587**, 98-102 (2020).
20. Shafer MER. Cross-Species Analysis of Single-Cell Transcriptomic Data. *Front Cell Dev Biol* **7**, (2019).

21. Wang X*, et al.* Comparative analysis of cell lineage differentiation during hepatogenesis in humans and mice at the single-cell transcriptome level. *Cell Res* **30**, 1109-1126 (2020).
