# Supplementary material for: Triiodothyronine (T3) promotes brown fat hyperplasia via thyroid hormone receptor α mediated adipocyte progenitor cell proliferation
Source: Nat Commun. 2022 Jun 13;13:3394. doi: 10.1038/s41467-022-31154-1 (PMC9192766; doi:10.1038/s41467-022-31154-1)
Supplement: Supplementary file 3 — Description of Additional Supplementary Files [file 41467_2022_31154_MOESM3_ESM.pdf]

Description of Additional Supplementary Files

File Name: Supplementary Data 1
Description: Differentially expressed genes between clusters as detected by scRNA-seq.

File Name: Supplementary Data 2.
Description: Differentially expressed genes between groups as detected by scRNA-seq.

File Name: Supplementary Data 3
Description: Gene Ontology (GO) analysis of the up-regulated genes in Group 5.

File Name: Supplementary Data 4
Description: Differentially expressed genes between states as detected by scRNA-seq.

File Name: Supplementary Data 5
Description: Gene Ontology (GO) analysis of the up-regulated genes in T3 group vs MMI group.

File Name: Supplementary Data 6
Description: Primer information.
