# Supplementary material for: Triiodothyronine (T3) promotes brown fat hyperplasia via thyroid hormone receptor α mediated adipocyte progenitor cell proliferation
Source: Nat Commun. 2022 Jun 13;13:3394. doi: 10.1038/s41467-022-31154-1 (PMC9192766; doi:10.1038/s41467-022-31154-1)
Supplement: Supplementary file 10 — Reporting Summary [file 41467_2022_31154_MOESM10_ESM.pdf]

nature portfolio

Corresponding author(s): Hao Ying, Jingjing Jiang

Last updated by author(s): May 25, 2022

Reporting Summary

Nature Portfolio wishes to improve the reproducibility of the work that we publish. This form provides structure for consistency and transparency in reporting. For further information on Nature Portfolio policies, see our Editorial Policies and the Editorial Policy Checklist.

Statistics

For all statistical analyses, confirm that the following items are present in the figure legend, table legend, main text, or Methods section.

n/a	Confirmed	
☐	☒	The exact sample size (*n*) for each experimental group/condition, given as a discrete number and unit of measurement
☐	☒	A statement on whether measurements were taken from distinct samples or whether the same sample was measured repeatedly
☐	☒	The statistical test(s) used AND whether they are one- or two-sided<br>*Only common tests should be described solely by name; describe more complex techniques in the Methods section.*
☒	☐	A description of all covariates tested
☐	☒	A description of any assumptions or corrections, such as tests of normality and adjustment for multiple comparisons
☐	☒	A full description of the statistical parameters including central tendency (e.g. means) or other basic estimates (e.g. regression coefficient) AND variation (e.g. standard deviation) or associated estimates of uncertainty (e.g. confidence intervals)
☐	☒	For null hypothesis testing, the test statistic (e.g. *F*, *t*, *r*) with confidence intervals, effect sizes, degrees of freedom and *P* value noted<br>*Give P values as exact values whenever suitable.*
☒	☐	For Bayesian analysis, information on the choice of priors and Markov chain Monte Carlo settings
☒	☐	For hierarchical and complex designs, identification of the appropriate level for tests and full reporting of outcomes
☐	☒	Estimates of effect sizes (e.g. Cohen's *d*, Pearson's *r*), indicating how they were calculated

Our web collection on statistics for biologists contains articles on many of the points above.

Software and code

Policy information about availability of computer code

Data collection	qPCR reactions were conducted using QuantStudio 6 (Thermo Fisher);<br>Microscopy pictures were acquired with LSM880NLO FLIM (Carl ZEISS) and REVOLVE (ECHO-labs);<br>Flow cytometry data were acquired with Gallios and Beckman Moflo Astrios (Beckman).<br>The single-cell RNA-seq libraries were sequenced on an Illumina HiSeq X Ten Illumina HiSeq4000 platform (Illumina). The Cell Ranger Single Cell Software Suite v.3.0 was used to de-multiplex individual cells, process UMIs, and count UMIs per gene, following standard pipeline.
Data analysis	GraphPad Prism version 8.0.2 was used to data analysis;<br>ZEN 2.3 (blue edition) software was used to acquisition of images from confocal microscope;<br>ImageJ v1.8.0 (National Institutes of Health) were used to Western blot densitometry analysis;<br>FlowJo_V10 were used to FACS analysis.<br>The on-line siRNA software (broadinstitute.org) were used for siRNA sequences.<br>The following R 3.5.1 package were used for the analysis of scRNA-seq data: Seurat (v 3.2.2), Monocle (v 2.18.0).<br>No custom code was used.

For manuscripts utilizing custom algorithms or software that are central to the research but not yet described in published literature, software must be made available to editors and reviewers. We strongly encourage code deposition in a community repository (e.g. GitHub). See the Nature Portfolio guidelines for submitting code & software for further information.

Data

Policy information about availability of data

All manuscripts must include a data availability statement. This statement should provide the following information, where applicable:

- Accession codes, unique identifiers, or web links for publicly available datasets
- A description of any restrictions on data availability
- For clinical datasets or third party data, please ensure that the statement adheres to our policy

Data supporting the findings of this study are available within the article and its Supplementary information files or from the corresponding author upon reasonable request. The scRNA-seq data generated in this study have been deposited in the Gene Expression Omnibus database under accession code GSE186188 (https://www.ncbi.nlm.nih.gov/geo/query/acc.cgi?&acc=GSE186188). The scRNA-seq data from other publications used in this study are available in the ArrayExpress under accession code E-MTAB-8564[https://www.ebi.ac.uk/arrayexpress/experiments/E-MTAB-8564/], and Sequence Read Archive under accession code SRX4074084[https://www.ncbi.nlm.nih.gov/sra/?term=SRX4074084%5d], SRX4074085[https://www.ncbi.nlm.nih.gov/sra/?term=SRX4074085], SRX4074088[https://www.ncbi.nlm.nih.gov/sra/?term=SRX4074088] and SRX4074089[https://www.ncbi.nlm.nih.gov/sra/?term=SRX4074089]. Source data are provided with this paper.

Field-specific reporting

Please select the one below that is the best fit for your research. If you are not sure, read the appropriate sections before making your selection.

☒ Life sciences ☐ Behavioural & social sciences ☐ Ecological, evolutionary & environmental sciences

For a reference copy of the document with all sections, see nature.com/documents/nr-reporting-summary-flat.pdf

Life sciences study design

All studies must disclose on these points even when the disclosure is negative.

Sample size	At least three biological replicates per group were collected to perform statistical testing. Detailed n is indicated in the figure legends. No statistical method was used to determine the sample size. The sample sizes are determined empirically, and are similar in size to most existing studies in the field.
Data exclusions	In the scRNA-seq experiment, cells with low quality were removed. Besides, no data or samples were excluded from the analysis.
Replication	Experiments were performed at least twice successfully to make sure similar results are reproducible, and representative data are shown. Details are indicated in Method.
Randomization	All samples/animals were randomly allocated to experimental groups and processed.
Blinding	Blinding strategy were employed whenever possible. Experimenters were blinded to group allocation for mouse-based experiments, FACS and IF. The research staff who performed the scRNA-sequencing library preparation had no knowledge about the sample characteristics. Cell-based experiments, western blotting and ChIP were not performed blind to load samples by order.

Behavioural & social sciences study design

All studies must disclose on these points even when the disclosure is negative.

Study description	*Briefly describe the study type including whether data are quantitative, qualitative, or mixed-methods (e.g. qualitative cross-sectional, quantitative experimental, mixed-methods case study).*
Research sample	*State the research sample (e.g. Harvard university undergraduates, villagers in rural India) and provide relevant demographic information (e.g. age, sex) and indicate whether the sample is representative. Provide a rationale for the study sample chosen. For studies involving existing datasets, please describe the dataset and source.*
Sampling strategy	*Describe the sampling procedure (e.g. random, snowball, stratified, convenience). Describe the statistical methods that were used to predetermine sample size OR if no sample-size calculation was performed, describe how sample sizes were chosen and provide a rationale for why these sample sizes are sufficient. For qualitative data, please indicate whether data saturation was considered, and what criteria were used to decide that no further sampling was needed.*
Data collection	*Provide details about the data collection procedure, including the instruments or devices used to record the data (e.g. pen and paper, computer, eye tracker, video or audio equipment) whether anyone was present besides the participant(s) and the researcher, and whether the researcher was blind to experimental condition and/or the study hypothesis during data collection.*
Timing	*Indicate the start and stop dates of data collection. If there is a gap between collection periods, state the dates for each sample cohort.*

Data exclusions | *If no data were excluded from the analyses, state so OR if data were excluded, provide the exact number of exclusions and the rationale behind them, indicating whether exclusion criteria were pre-established.*

Non-participation | *State how many participants dropped out/declined participation and the reason(s) given OR provide response rate OR state that no participants dropped out/declined participation.*

Randomization | *If participants were not allocated into experimental groups, state so OR describe how participants were allocated to groups, and if allocation was not random, describe how covariates were controlled.*

Ecological, evolutionary & environmental sciences study design

All studies must disclose on these points even when the disclosure is negative.

Study description | *Briefly describe the study. For quantitative data include treatment factors and interactions, design structure (e.g. factorial, nested, hierarchical), nature and number of experimental units and replicates.*

Research sample | *Describe the research sample (e.g. a group of tagged Passer domesticus, all Stenocereus thurberi within Organ Pipe Cactus National Monument), and provide a rationale for the sample choice. When relevant, describe the organism taxa, source, sex, age range and any manipulations. State what population the sample is meant to represent when applicable. For studies involving existing datasets, describe the data and its source.*

Sampling strategy | *Note the sampling procedure. Describe the statistical methods that were used to predetermine sample size OR if no sample-size calculation was performed, describe how sample sizes were chosen and provide a rationale for why these sample sizes are sufficient.*

Data collection | *Describe the data collection procedure, including who recorded the data and how.*

Timing and spatial scale | *Indicate the start and stop dates of data collection, noting the frequency and periodicity of sampling and providing a rationale for these choices. If there is a gap between collection periods, state the dates for each sample cohort. Specify the spatial scale from which the data are taken*

Data exclusions | *If no data were excluded from the analyses, state so OR if data were excluded, describe the exclusions and the rationale behind them, indicating whether exclusion criteria were pre-established.*

Reproducibility | *Describe the measures taken to verify the reproducibility of experimental findings. For each experiment, note whether any attempts to repeat the experiment failed OR state that all attempts to repeat the experiment were successful.*

Randomization | *Describe how samples/organisms/participants were allocated into groups. If allocation was not random, describe how covariates were controlled. If this is not relevant to your study, explain why.*

Blinding | *Describe the extent of blinding used during data acquisition and analysis. If blinding was not possible, describe why OR explain why blinding was not relevant to your study.*

Did the study involve field work? ☐ Yes ☐ No

Field work, collection and transport

Field conditions | *Describe the study conditions for field work, providing relevant parameters (e.g. temperature, rainfall).*

Location | *State the location of the sampling or experiment, providing relevant parameters (e.g. latitude and longitude, elevation, water depth).*

Access & import/export | *Describe the efforts you have made to access habitats and to collect and import/export your samples in a responsible manner and in compliance with local, national and international laws, noting any permits that were obtained (give the name of the issuing authority, the date of issue, and any identifying information).*

Disturbance | *Describe any disturbance caused by the study and how it was minimized.*

Reporting for specific materials, systems and methods

We require information from authors about some types of materials, experimental systems and methods used in many studies. Here, indicate whether each material, system or method listed is relevant to your study. If you are not sure if a list item applies to your research, read the appropriate section before selecting a response.

Materials & experimental systems

n/a	Involved in the study	
☐	☒	Antibodies
☐	☒	Eukaryotic cell lines
☒	☐	Palaeontology and archaeology
☐	☒	Animals and other organisms
☐	☒	Human research participants
☒	☐	Clinical data
☒	☐	Dual use research of concern

Methods

n/a	Involved in the study	
☐	☐	ChIP-seq
☐	☒	Flow cytometry
☐	☐	MRI-based neuroimaging

Antibodies

Antibodies used

For Western blot, the following antibodies were used:
UCP1 (ABCAM, ab10983); MYC (Cell Signaling Technology, 5605); HK2 (Cell SignalingTechnology, 2867); HSP90 (Cell Signaling Technology, 4874); GAPDH (Cell Signaling Technology, 5174); Tubulin (Cell Signaling Technology, 3873); Tyrosine hydroxylase (Millipore, AB152).
For immunofluorescent staining, the following antibodies were used:
UCP1 (ABCAM, ab10983); anti-red fluorescent protein (Rockland, 000-001-379); m-DPP4/CD26 (R&D, AF954-SP); mPDGFRα (R&D, AF1062-SP);secondary antibodies Alexa Fluor® 594 conjugated goat antibody to rabbit IgG (Invitrogen, A-11012); Alexa Fluor® 647 conjugated donkey antibody to goat IgG (Invitrogen, A-21447); BODIPY 493/503 (Invitrogen, D3922); Click-iT® Plus EdU Alexa Fluor® 488 Imaging Kit (Invitrogen, C10337).
For immunohistochemical staining, the following antibodies were used:
UCP1 (ABCAM, ab10983).
For FACS analysis, the following antibodies were used:
PE/Cy7 anti-human CD90 (Biolegend, 328123); FITC anti-human CD31 (Invitrogen, 11-0319-41); APC/Cy7 anti-human CD45 (Biolegend, 368515); FITC anti-mouse CD31 (Biolegend, 102406); PE anti mouse Ly-6A/E (Sca1) (Invitrogen, 12-5981-82); PE/Cy7 anti-mouse CD31 (Biolegend, 102524); APC/Cy7 anti-mouse CD45 (Biolegend, 103116); APC anti-mouse CD26 (DPP-4) (Biolegend, 137807); PE/Cy7 anti-mouse CD54 (ICAM-1)(Biolegend, 116121); APC/Cyanine7 anti-mouse CD31 (Biolegend, 102440); Alexa Fluor® 488 anti-mouse/human Ki-67 (Biolegend, 151204); FITC anti-mouse Ly-6A/E (Sca-1) (Invitrogen, 11-5981-82); FITC BrdU Flow Kit (BD Pharmingen, 559619); anti-mouse CD16/32 antibody (Invitrogen, 14-0161-85).
For ChIP assay, the following antibodies were used:
HA-Tag (Cell Signaling Technology, 3724).

Validation

All antibodies are commercially available and were validated as follows:
UCP1: https://www.abcam.cn/ucp1-antibody-ab10983.html;
MYC: https://www.cellsignal.cn/products/primary-antibodies/c-myc-d84c12-rabbit-mab/5605?site-search-type=Products&N=4294956287&Ntt=5605&fromPage=plp&_requestid=8688528;
HK2: https://www.cellsignal.cn/products/primary-antibodies/hexokinase-ii-c64g5-rabbit-mab/2867?site-search-type=Products&N=4294956287&Ntt=c64g5&fromPage=plp;
HSP90: https://www.cellsignal.cn/products/primary-antibodies/hsp90-antibody/4874?site-search-type=Products&N=4294956287&Ntt=4874&fromPage=plp&_requestid=8688796;
GAPDH: https://www.cellsignal.cn/products/primary-antibodies/a-tubulin-dm1a-mouse-mab/3873?site-search-type=Products&N=4294956287&Ntt=3873&fromPage=plp&_requestid=8689678;
Tubulin: https://www.cellsignal.cn/products/primary-antibodies/a-tubulin-dm1a-mouse-mab/3873?site-search-type=Products&N=4294956287&Ntt=3873&fromPage=plp&_requestid=1502965;
Tyrosine hydroxylase: https://www.sigmaaldrich.cn/CN/en/product/mm/ab152;
anti-red fluorescent protein: https://www.rockland.com/categories/proteins-and-peptides/recombinant-red-fluorescent-protein-rfp-control-000-001-379/;
m-DPP4/CD26: https://www.rndsystems.com/cn/products/mouse-dppiv-cd26-antibody_af954;
mPDGFRα: https://www.rndsystems.com/cn/products/mouse-pdgf-ralpha-antibody_af1062;
Alexa Fluor® 594 conjugated goat antibody to rabbit IgG : https://www.thermofisher.cn/cn/zh/antibody/product/Goat-anti-Rabbit-IgG-H-L-Cross-Adsorbed-Secondary-Antibody-Polyclonal/A-11012;
Alexa Fluor® 647 conjugated donkey antibody to goat IgG: https://www.thermofisher.cn/cn/zh/antibody/product/Donkey-anti-Goat-IgG-H-L-Cross-Adsorbed-Secondary-Antibody-Polyclonal/A-21447?adobe_mc=MCMID%7C63218995283614020031223695742181091705%7CMCAID%3D2F7CA62B8515B92D-60000953D39F041D%7CMCORGID%3D5B135A0C5370E6B40A490D44@AdobeOrg%7CTS%3D1614293705;
BODIPY 493/503: https://www.thermofisher.cn/order/catalog/product/D3922?SID=srch-hj-D3922;
Click-iT® Plus EdU Alexa Fluor® 488 Imaging Kit: https://www.thermofisher.cn/order/catalog/product/C10337?SID=srch-srp-C10337;
PE/Cy7 anti-human CD90: https://www.biolegend.com/en-us/products/pe-cyanine7-anti-human-cd90-thy1-antibody-8282;
FITC anti-human CD31: https://www.thermofisher.cn/cn/zh/antibody/product/CD31-PECAM-1-Antibody-clone-WM-59-WM59-Monoclonal/11-0319-41;
APC/Cy7 anti-human CD45: https://www.biolegend.com/en-us/products/apc-cyanine7-anti-human-cd45-antibody-12400;
FITC anti-mouse CD31: https://www.biolegend.com/en-us/products/fitc-anti-mouse-cd31-antibody-120;
PE anti mouse Ly-6A/E (Sca1): https://www.thermofisher.cn/cn/zh/antibody/product/Ly-6A-E-Sca-1-Antibody-clone-D7-Monoclonal/12-5981-82;
PE/Cy7 anti-mouse CD31: https://www.biolegend.com/en-us/products/pe-cyanine7-anti-mouse-cd31-antibody-12996;
APC/Cy7 anti-mouse CD45: https://www.biolegend.com/en-us/products/apc-cyanine7-anti-mouse-cd45-antibody-2530;
APC anti-mouse CD26 (DPP-4): https://www.biolegend.com/en-us/products/apc-anti-mouse-cd26-dpp-4-antibody-6947;
PE/Cy7 anti-mouse CD54 (ICAM-1): https://www.biolegend.com/en-us/products/pe-cyanine7-anti-mouse-cd54-antibody-14759;
APC/Cyanine7 anti-mouse CD31: https://www.biolegend.com/en-us/products/apc-cyanine7-anti-mouse-cd31-antibdoy-18889;

Alexa Fluor® 488 anti-mouse/human Ki-67: https://www.biolegend.com/en-us/products/alexa-fluor-488-anti-mouse-human-ki-67-antibody-12889;
FITC anti-mouse Ly-6A/E (Sca-1): https://www.thermofisher.cn/cn/zh/antibody/product/Ly-6A-E-Sca-1-Antibody-clone-D7-Monoclonal/11-5981-82;
FITC BrdU Flow Kit: https://www.bdbiosciences.com/en-eu/products/reagents/flow-cytometry-reagents/research-reagents/panels-multicolor-cocktails-ruo/fitc-brdu-flow-kit.559619;
anti-mouse CD16/32 antibody: https://www.thermofisher.cn/cn/zh/antibody/product/CD16-CD32-Antibody-clone-93-Monoclonal/14-0161-85;
HA-Tag: https://www.cellsignal.cn/products/primary-antibodies/ha-tag-c29f4-rabbit-mab/3724?site-search-type=Products&N=4294956287&Ntt=3724&fromPage=plp&_requestid=8701061.

Eukaryotic cell lines

Policy information about cell lines

Cell line source(s)	BAC (brown adipocyte precursor cell line) were obtained from our own lab. HEK-293T cell line was obtained from American Type Culture Collection (ATCC).
Authentication	BACs were authenticated by morphology, gene expression profile and differentiation potentiality.
Mycoplasma contamination	Cell lines were tested negative for mycoplasma.
Commonly misidentified lines (See ICLAC register)	No commonly misidentified cell lines were involved in this study.

Palaeontology and Archaeology

Specimen provenance	*Provide provenance information for specimens and describe permits that were obtained for the work (including the name of the issuing authority, the date of issue, and any identifying information). Permits should encompass collection and, where applicable, export.*
Specimen deposition	*Indicate where the specimens have been deposited to permit free access by other researchers.*
Dating methods	*If new dates are provided, describe how they were obtained (e.g. collection, storage, sample pretreatment and measurement), where they were obtained (i.e. lab name), the calibration program and the protocol for quality assurance OR state that no new dates are provided.*

☐ Tick this box to confirm that the raw and calibrated dates are available in the paper or in Supplementary Information.

Ethics oversight	*Identify the organization(s) that approved or provided guidance on the study protocol, OR state that no ethical approval or guidance was required and explain why not.*

Note that full information on the approval of the study protocol must also be provided in the manuscript.

Animals and other organisms

Policy information about studies involving animals; ARRIVE guidelines recommended for reporting animal research

Laboratory animals	Male wild-type or genetically engineered mice with the C57BL/6J background at 8-12 weeks old have been used in this study. Unless otherwise stated, mice used were male C57BL/6J, and housed under 12-h/12-h light/dark cycle and humidity 50-60% with free access to food and water. TRαFloxed mice were purchased from the Jackson Laboratory. TRβFloxed mice were generated in the Shanghai Biomodel Organism Science & Technology Development. Rosa26-tdTomato reporter mice were purchased from the Jackson Laboratory. Myf5-Cre mice were purchased from the Jackson Laboratory. UCP1-Cre mice were gifts from Prof. Kong Xingxing (UCLA).
Wild animals	The study did not involve wild animals.
Field-collected samples	The study did not involve samples collected from the field.
Ethics oversight	Experiments involving mice were all in accordance with institutional guidelines for the care and use of animals. Animal protocols were approved by the Ethics Committee of Shanghai Institute of Nutrition and Health, Chinese Academy of Sciences (2015-AN-12, 2016-AN-1, SIBS-2019-YH-1).

Note that full information on the approval of the study protocol must also be provided in the manuscript.

Human research participants

Policy information about studies involving human research participants

Population characteristics	Sixteen patients (8 males and 8 females, aged between 29-68 years old) were recruited prior to thyroidectomy due to papillary thyroid cancer. All patients had normal thyroid function, i.e., free T3, free T4, and TSH all in normal range.
Recruitment	Patients with papillary thyroid cancer, aged between 18 and 80 years old, were eligible for participation. The exclusion criteria included: pregnancy of female; diabetes; presence of other severe diseases involving heart, lung, liver, kidney, etc;

	any other type of cancer or history of cancer besides papillary thyroid cancer; hereditary diseases; autoimmune diseases; use of any type of steroids (oral, inhaled, or topical) in the past three months. Patients were randomly recruited from the Department of General Surgery, Zhongshan Hospital, Fudan University. Adipose tissues were collected from the neck by an experienced surgeon during thyroidectomy. No compensation was paid for participation in this study.
Ethics oversight	The study protocol was approved by the Ethics Committee of Zhongshan Hospital, Fudan University. Informed consent was obtained from all participants.

Note that full information on the approval of the study protocol must also be provided in the manuscript.

Clinical data

Policy information about clinical studies

All manuscripts should comply with the ICMJE guidelines for publication of clinical research and a completed CONSORT checklist must be included with all submissions.

Clinical trial registration	*Provide the trial registration number from ClinicalTrials.gov or an equivalent agency.*
Study protocol	*Note where the full trial protocol can be accessed OR if not available, explain why.*
Data collection	*Describe the settings and locales of data collection, noting the time periods of recruitment and data collection.*
Outcomes	*Describe how you pre-defined primary and secondary outcome measures and how you assessed these measures.*

Dual use research of concern

Policy information about dual use research of concern

Hazards

Could the accidental, deliberate or reckless misuse of agents or technologies generated in the work, or the application of information presented in the manuscript, pose a threat to:

No	Yes	
☐	☐	Public health
☐	☐	National security
☐	☐	Crops and/or livestock
☐	☐	Ecosystems
☐	☐	Any other significant area

Experiments of concern

Does the work involve any of these experiments of concern:

No	Yes	
☐	☐	Demonstrate how to render a vaccine ineffective
☐	☐	Confer resistance to therapeutically useful antibiotics or antiviral agents
☐	☐	Enhance the virulence of a pathogen or render a nonpathogen virulent
☐	☐	Increase transmissibility of a pathogen
☐	☐	Alter the host range of a pathogen
☐	☐	Enable evasion of diagnostic/detection modalities
☐	☐	Enable the weaponization of a biological agent or toxin
☐	☐	Any other potentially harmful combination of experiments and agents

ChIP-seq

Data deposition

☐ Confirm that both raw and final processed data have been deposited in a public database such as GEO.

☐ Confirm that you have deposited or provided access to graph files (e.g. BED files) for the called peaks.

Data access links<br>*May remain private before publication.*	*For "Initial submission" or "Revised version" documents, provide reviewer access links. For your "Final submission" document, provide a link to the deposited data.*
Files in database submission	*Provide a list of all files available in the database submission.*

Genome browser session (e.g. UCSC) | *Provide a link to an anonymized genome browser session for "Initial submission" and "Revised version" documents only, to enable peer review. Write "no longer applicable" for "Final submission" documents.*

Methodology

Replicates	*Describe the experimental replicates, specifying number, type and replicate agreement.*
Sequencing depth	*Describe the sequencing depth for each experiment, providing the total number of reads, uniquely mapped reads, length of reads and whether they were paired- or single-end.*
Antibodies	*Describe the antibodies used for the ChIP-seq experiments; as applicable, provide supplier name, catalog number, clone name, and lot number.*
Peak calling parameters	*Specify the command line program and parameters used for read mapping and peak calling, including the ChIP, control and index files used.*
Data quality	*Describe the methods used to ensure data quality in full detail, including how many peaks are at FDR 5% and above 5-fold enrichment.*
Software	*Describe the software used to collect and analyze the ChIP-seq data. For custom code that has been deposited into a community repository, provide accession details.*

Flow Cytometry

Plots

Confirm that:

- [x] The axis labels state the marker and fluorochrome used (e.g. CD4-FITC).
- [ ] The axis scales are clearly visible. Include numbers along axes only for bottom left plot of group (a 'group' is an analysis of identical markers).
- [x] All plots are contour plots with outliers or pseudocolor plots.
- [x] A numerical value for number of cells or percentage (with statistics) is provided.

Methodology

Sample preparation	SVF cells of iBAT from mice were isolated using 1mg/mL Collagenase Type 1 (Sigma) digestion, followed by density separation. Briefly, iBAT depots were collected and minced into pieces, followed by digestion at 37°C for 40 minutes. Cell suspensions were filtered through 70 µm filters and centrifuged at 800 g for 5 minutes. SVF cells were incubated in red blood cell lysis buffer for 5 minutes on ice, centrifuged at 800 g for 5 minutes at 4°C, and then resuspended in staining buffer (0.1% BSA in PBS). Dead cells were stained with LIVE/DEAD™ Fixable Violet Dead Cell Stain Kit (Invitrogen), then gated out in analysis. Anti-mouse CD16/32 antibody (Invitrogen) was used for murine cells to block nonspecific binding before surface staining. Afterwards, cells were washed once with PBS followed by staining with surface antibodies.
Instrument	Gallios and Beckman Moflo Astrios EQ.
Software	FlowJo were used to FACS analysis.
Cell population abundance	We stained isolated cells by FACS with CD45, CD31 and Sca1 antibodies which is canonical marker of APCs. Our flow cytometry analysis revealed that we could obtain ~17% APCs in live SVF cells of iBAT from male C57BL/6J mice.
Gating strategy	For analysis of APCs, we set appropriate FSC vs SSC gates to exclude debris and cell aggregates. Staining the dead cells, we draw a gate around the live cells population. Then, we draw a gate around the CD45– population. Next, we draw a gate around the CD31–Sca1+ population identified as APCs.

- [x] Tick this box to confirm that a figure exemplifying the gating strategy is provided in the Supplementary Information.

Magnetic resonance imaging

Experimental design

Design type	*Indicate task or resting state; event-related or block design.*
Design specifications	*Specify the number of blocks, trials or experimental units per session and/or subject, and specify the length of each trial or block (if trials are blocked) and interval between trials.*
Behavioral performance measures	*State number and/or type of variables recorded (e.g. correct button press, response time) and what statistics were used to establish that the subjects were performing the task as expected (e.g. mean, range, and/or standard deviation across subjects).*

Acquisition

Imaging type(s)	*Specify: functional, structural, diffusion, perfusion.*
Field strength	*Specify in Tesla*
Sequence & imaging parameters	*Specify the pulse sequence type (gradient echo, spin echo, etc.), imaging type (EPI, spiral, etc.), field of view, matrix size, slice thickness, orientation and TE/TR/flip angle.*
Area of acquisition	*State whether a whole brain scan was used OR define the area of acquisition, describing how the region was determined.*

Diffusion MRI ☐ Used ☐ Not used

Preprocessing

Preprocessing software	*Provide detail on software version and revision number and on specific parameters (model/functions, brain extraction, segmentation, smoothing kernel size, etc.).*
Normalization	*If data were normalized/standardized, describe the approach(es): specify linear or non-linear and define image types used for transformation OR indicate that data were not normalized and explain rationale for lack of normalization.*
Normalization template	*Describe the template used for normalization/transformation, specifying subject space or group standardized space (e.g. original Talairach, MNI305, ICBM152) OR indicate that the data were not normalized.*
Noise and artifact removal	*Describe your procedure(s) for artifact and structured noise removal, specifying motion parameters, tissue signals and physiological signals (heart rate, respiration).*
Volume censoring	*Define your software and/or method and criteria for volume censoring, and state the extent of such censoring.*

Statistical modeling & inference

Model type and settings	*Specify type (mass univariate, multivariate, RSA, predictive, etc.) and describe essential details of the model at the first and second levels (e.g. fixed, random or mixed effects; drift or auto-correlation).*
Effect(s) tested	*Define precise effect in terms of the task or stimulus conditions instead of psychological concepts and indicate whether ANOVA or factorial designs were used.*

Specify type of analysis: ☐ Whole brain ☐ ROI-based ☐ Both

Statistic type for inference (See Eklund et al. 2016)	*Specify voxel-wise or cluster-wise and report all relevant parameters for cluster-wise methods.*
Correction	*Describe the type of correction and how it is obtained for multiple comparisons (e.g. FWE, FDR, permutation or Monte Carlo).*

Models & analysis

n/a	Involved in the study
☐	☐ Functional and/or effective connectivity
☐	☐ Graph analysis
☐	☐ Multivariate modeling or predictive analysis

Functional and/or effective connectivity	*Report the measures of dependence used and the model details (e.g. Pearson correlation, partial correlation, mutual information).*
Graph analysis	*Report the dependent variable and connectivity measure, specifying weighted graph or binarized graph, subject- or group-level, and the global and/or node summaries used (e.g. clustering coefficient, efficiency, etc.).*
Multivariate modeling and predictive analysis	*Specify independent variables, features extraction and dimension reduction, model, training and evaluation metrics.*
